# Supplementary material for: The SPN-4 Rbfox RNA-binding protein selects maternal mRNAs for CCR4-NOT-dependent clearance in early Caenorhabditis elegans embryos
Source: Development. 2026 Jun 4;153(11):dev205295. doi: 10.1242/dev.205295 (PMC13286378; doi:10.1242/dev.205295)
Supplement: Supplementary information [file develop-153-205295-s1.pdf]

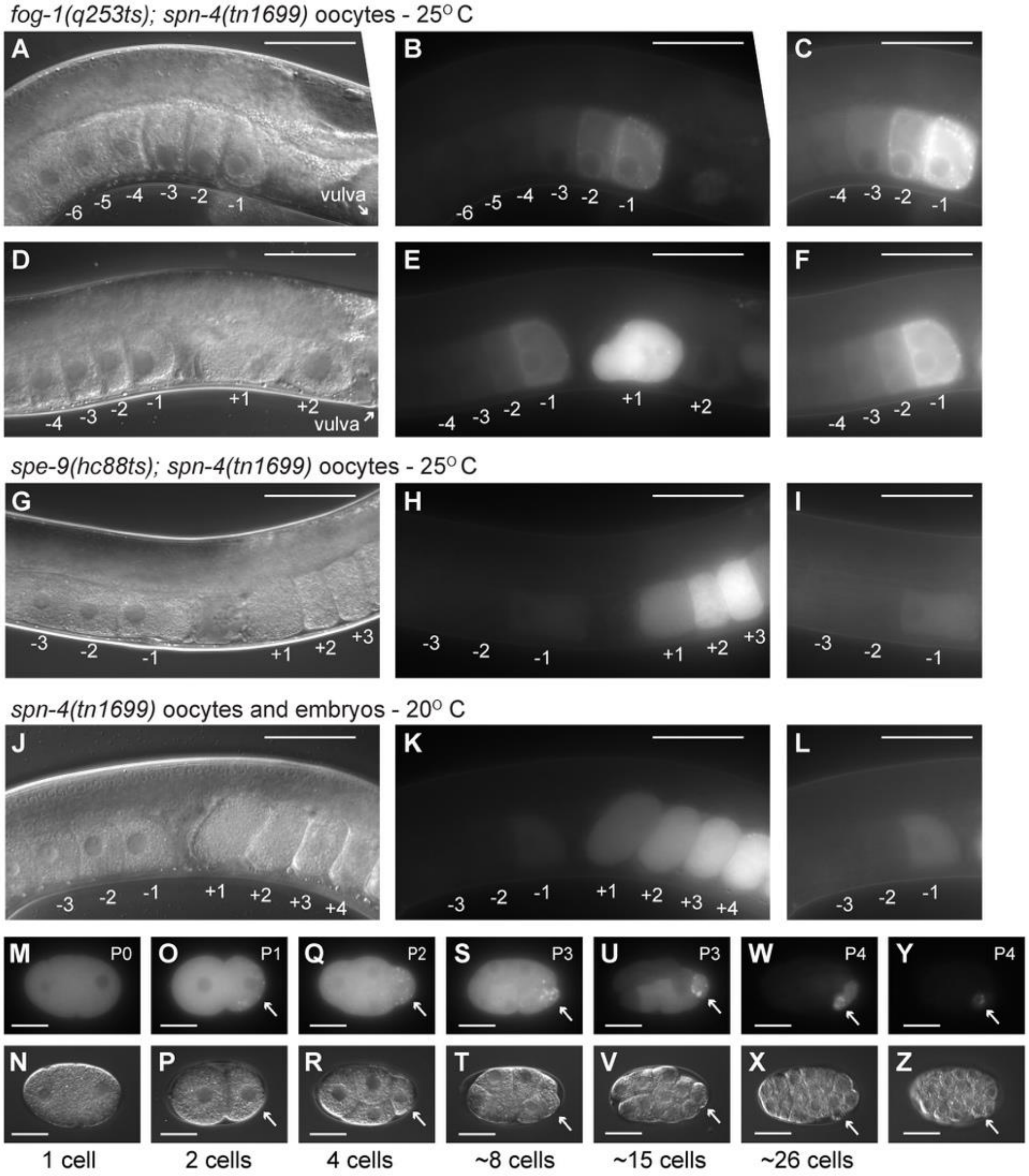

Fig. S1. Expression of SPN-4::GFP. (Related to Fig. 1 and Table S1). DIC (A,D,G,J,N,P,R,T,V,X,Z) and fluorescent micrographs (B,C,E,F,H,I,K,L,M,O,Q,S,U,W,Y) of SPN-4::GFP expression in the oogenic germline (A-L) and embryos (M-Z). The *fog-1(q253*ts*); spn-4(tn1699)* and *spe-9(hc88*ts*); spn-4(tn1699)* strains, which were used for the SPN-4 immunopurifications, were grown at 25°C. Panels (B,E,H,K) used 24 msec exposures with 70% SOLA light source intensity, whereas panels (C,F,I,L) used a longer exposure (80 msec at 80% SOLA light source intensity) to illustrate that SPN-4::GFP is more abundant in embryos and ovulated oocytes than it is in proximal oocytes in the gonad arm. In the embryo, SPN-4::GFP is present during the stages during which early mRNA clearance is observed; however, SPN-4::GFP becomes progressively restricted to the primordial germ cell P4. Bars, 50 μm (A-L); 20 μm (M-Z).

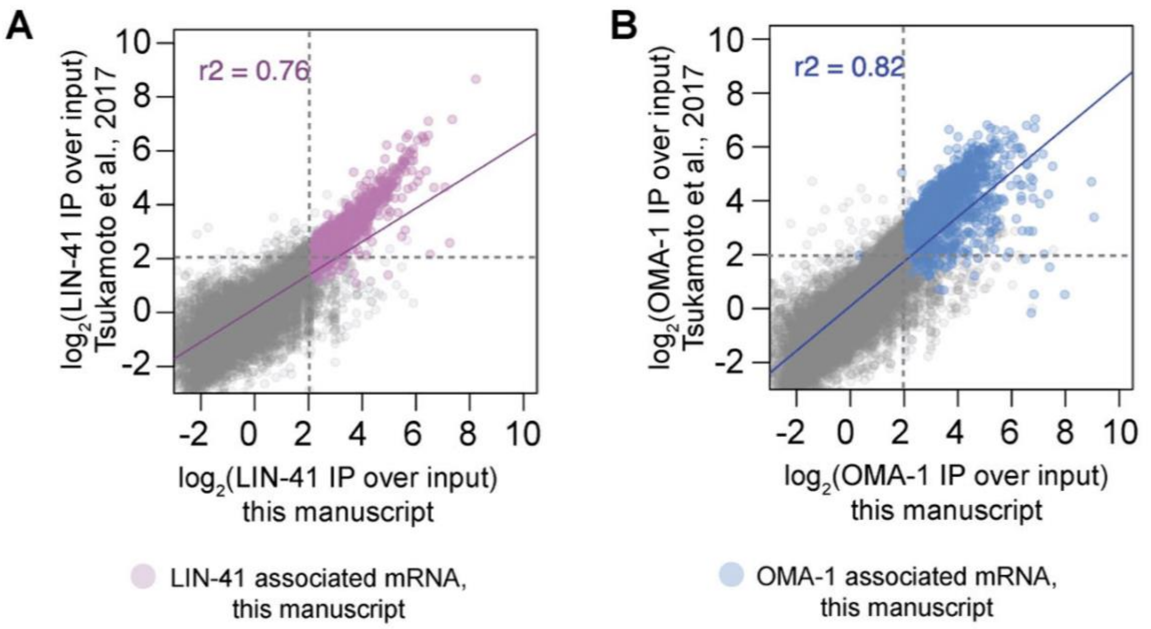

Fig. S2. RNA-binding protein immunopurifications from fertilization-defective strains. (Related to Fig. 1 and Table S1). In this study, SPN-4-, LIN-41- and OMA-1-associated mRNAs were sequenced using a low-input RNA-seq library whereas Tsukamoto et al. (2017) prepared sequencing libraries directly from the recovered mRNA for the analysis of LIN-41- and OMA-1-associated mRNAs (see Fig. 1 in the main text) (Tsukamoto et al., 2017). (A,B) Scatter plots comparing the log_2 values for immunopurified RNA over input for Tsukamoto et al. (2017) on the y-axis to results from this study (x-axis) for LIN-41 (A) and OMA-1 (B). Pearson correlation was calculated to include all points ("everything") using the cor function in R stats.

Development • Supplementary information

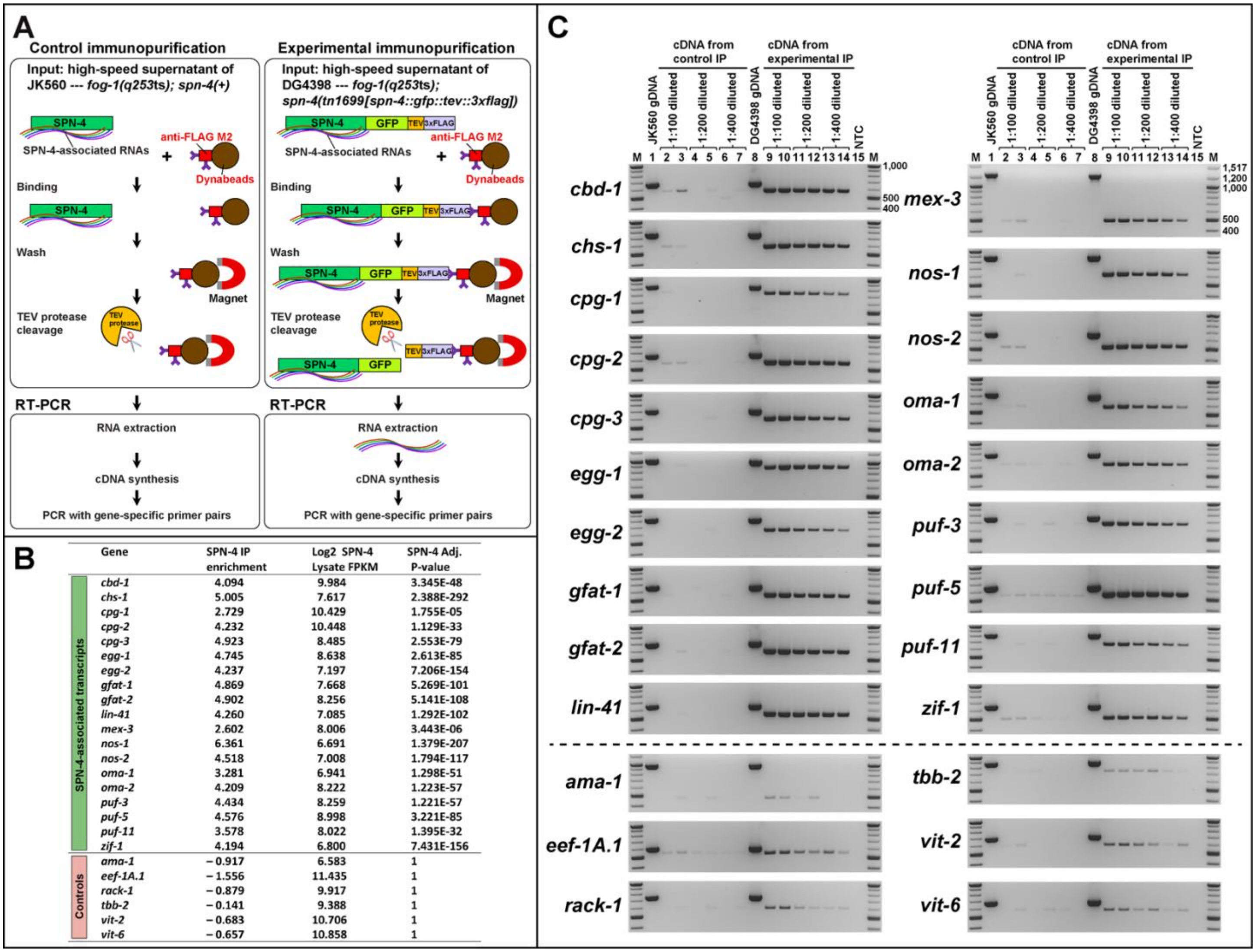

Gene	SPN-4 IP enrichment	Log2 SPN-4 Lysate FPKM	SPN-4 Adj. P-value
cbd-1	4.094	9.984	3.345E-48
chs-1	5.005	7.617	2.388E-292
cpg-1	2.729	10.429	1.755E-05
cpg-2	4.232	10.448	1.129E-33
cpg-3	4.923	8.485	2.553E-79
egg-1	4.745	8.638	2.613E-85
egg-2	4.237	7.197	7.206E-154
gfat-1	4.869	7.668	5.269E-101
gfat-2	4.902	8.256	5.141E-108
lin-41	4.260	7.085	1.292E-102
mex-3	2.602	8.006	3.443E-06
nos-1	6.361	6.691	1.379E-207
nos-2	4.518	7.008	1.794E-117
oma-1	3.281	6.941	1.298E-51
oma-2	4.209	8.222	1.223E-57
puf-3	4.434	8.259	1.221E-57
puf-5	4.576	8.998	3.221E-85
puf-11	3.578	8.022	1.395E-32
zif-1	4.194	6.800	7.431E-156
ama-1	−0.917	6.583	1
eef-1A.1	−1.556	11.435	1
rack-1	−0.879	9.917	1
tbb-2	−0.141	9.388	1
vit-2	−0.683	10.706	1
vit-6	−0.657	10.858	1

Fig. S3. Control for the specificity of the SPN-4 immunopurifications. (Related to Fig. 1 and Table S1). (A) Schematic of SPN-4 immunopurifications (IPs) from DG4398 *fog-1(q253*ts*); spn-4(tn1699[spn-4::gfp::tev::3xflag])* and the JK560 *fog-1(q253*ts*)* control. Eluted mRNA was analyzed by reverse transcription polymerase chain reaction (RT-PCR). (B) Data taken from Table S1 for the 19 SPN-4-associated transcripts and the six controls analyzed. (C) Analysis of RT-PCR products on ethidium bromide-stained 1.5% agarose gels. The cDNA dilutions shown were analyzed, as were genomic DNA (gDNA) and a no-template control (NTC). Non-specific binding to the control column was negligible (lanes 2-7). All SPN-4-associated transcripts were efficiently recovered in the experimental. The controls, *ama-1*, *eef-1A.1*, *rack-1*, *tbb-2*, *vit-2* and *vit-6,* which are among the most abundant *C. elegans* transcripts, were more prevalent in the experimental than the control, likely owing to non-specific absorption to the bound SPN-4 RNPs. However, RNA-sequencing shows that these transcripts are not significantly enriched compared to the starting lysate (Table S1 and panel B). The results of two technical replicates are shown in adjacent lanes for each dilution. The DNA size markers (M) used the 100-bp DNA ladder (New England Biolabs).

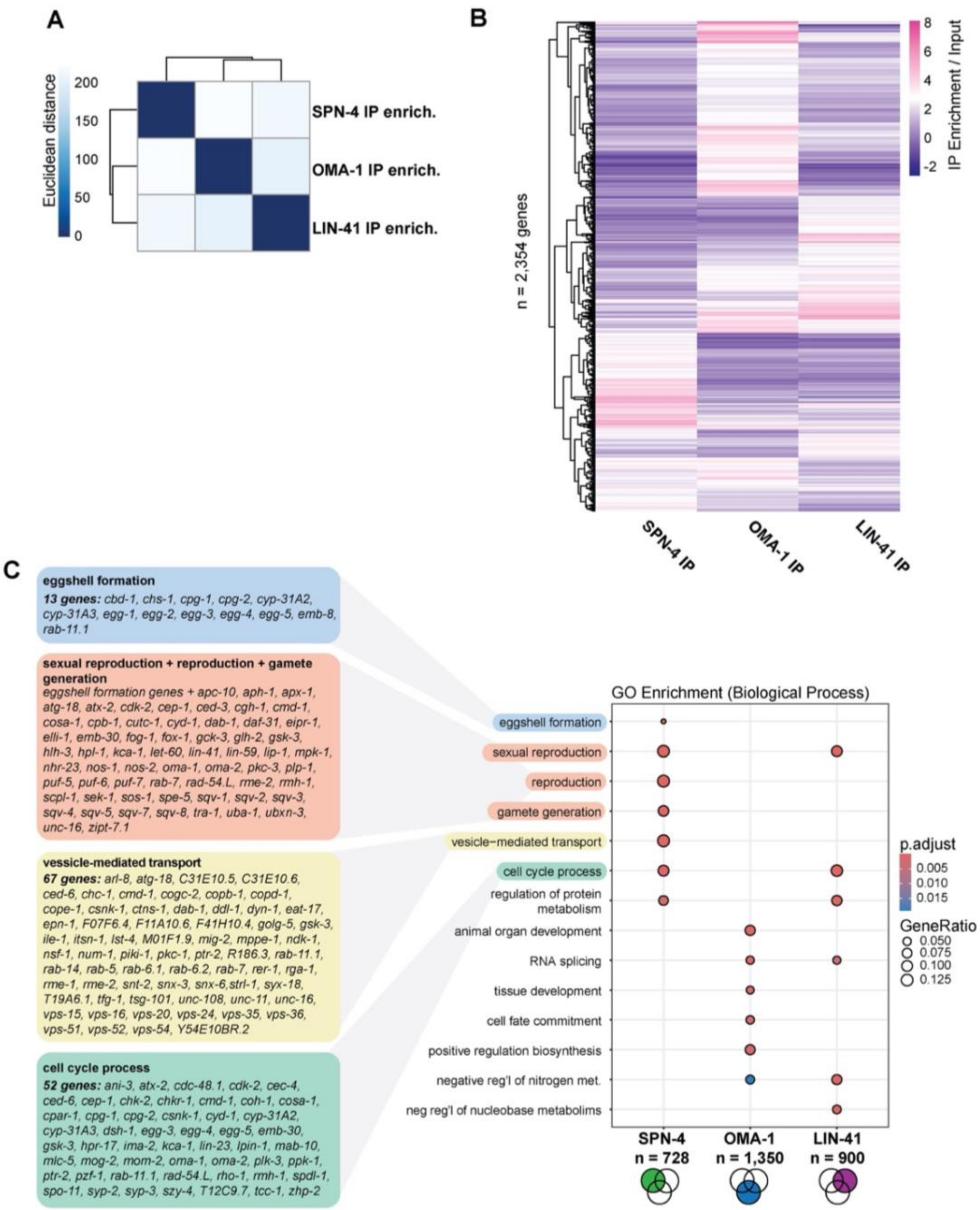

Fig. S4. Characteristics of SPN-4-, OMA-1- and LIN-41-associated transcripts. (Related to Fig. 1, Table S1 and Table S4). (A) This plot illustrates the relationship between mRNAs captured by the three different immunopurification assays. Transcripts were filtered for a minimum expression level (mean $\log_2(-2.5)$) and culled of cTel and 21U gene entries to yield 16,917 genes. Euclidean distance was calculated, clustered and plotted. Darker shading represents a more similar relationship. (B) Transcripts associated with at least one of the three RNA-binding proteins were plotted as a heatmap. Distance metrics were calculated using the Euclidean method and were clustered using the complete method, without applying z-scoring. (C) Gene Ontology (GO) terms enriched in the sets of SPN-4-, LIN-41- or OMA-1-associated transcripts are tabulated as dotplots in which terms shared across gene sets are comparable across a given row. The exact genes driving each GO term are listed and color-matched to their respective terms.

Development • Supplementary information

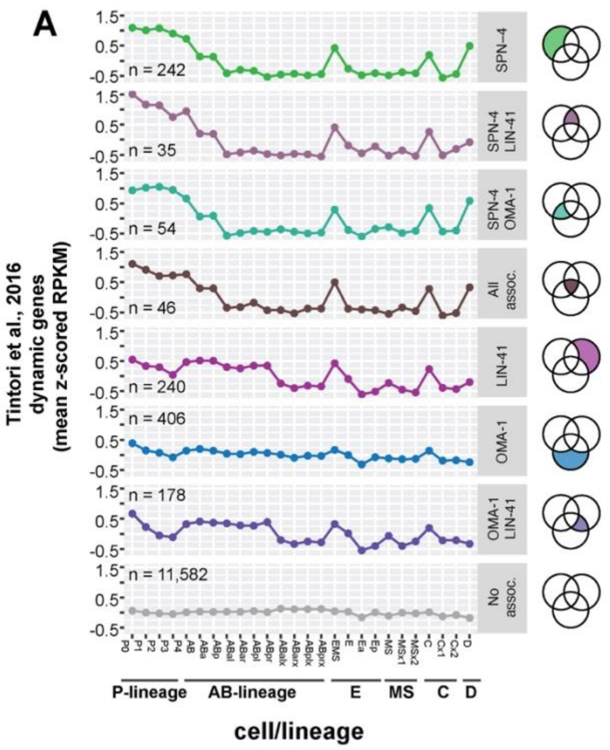

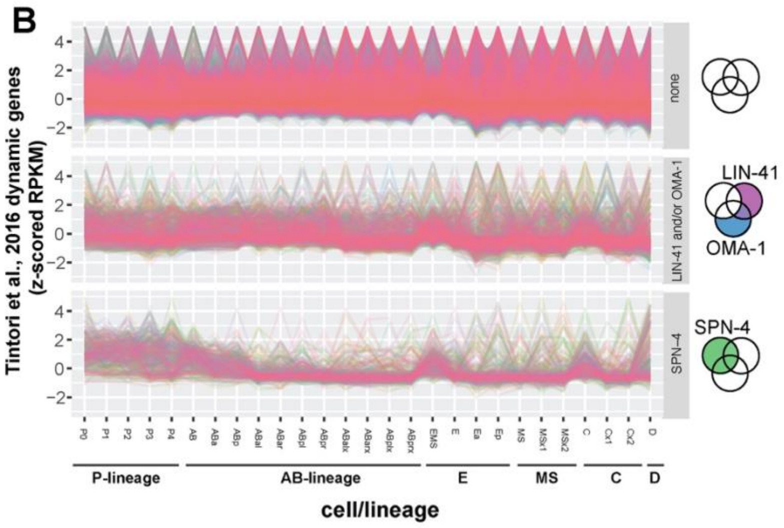

Fig. S5. Transcriptome profiles of genes through embryogenesis. (Related to Fig. 2). (A) Plots of mean z-scored transcript abundance for dynamically changing genes across development. Transcript abundance is plotted for each cell within each lineage as measured by single-cell RNA-seq (Tintori et al., 2016) grouped by association with SPN-4, LIN-41 and/or OMA-1. (B) The same data as in (A) but categorized into three main categories (SPN-4 association, OMA-1 and LIN-41 associated but not SPN-4 associated, or association with no assayed RNA-binding proteins). In lieu of mean intensity, z-scored intensities are plotted as transparent line plots for each transcript to illustrate the variance in each category. Colors are assigned randomly.

Development • Supplementary information

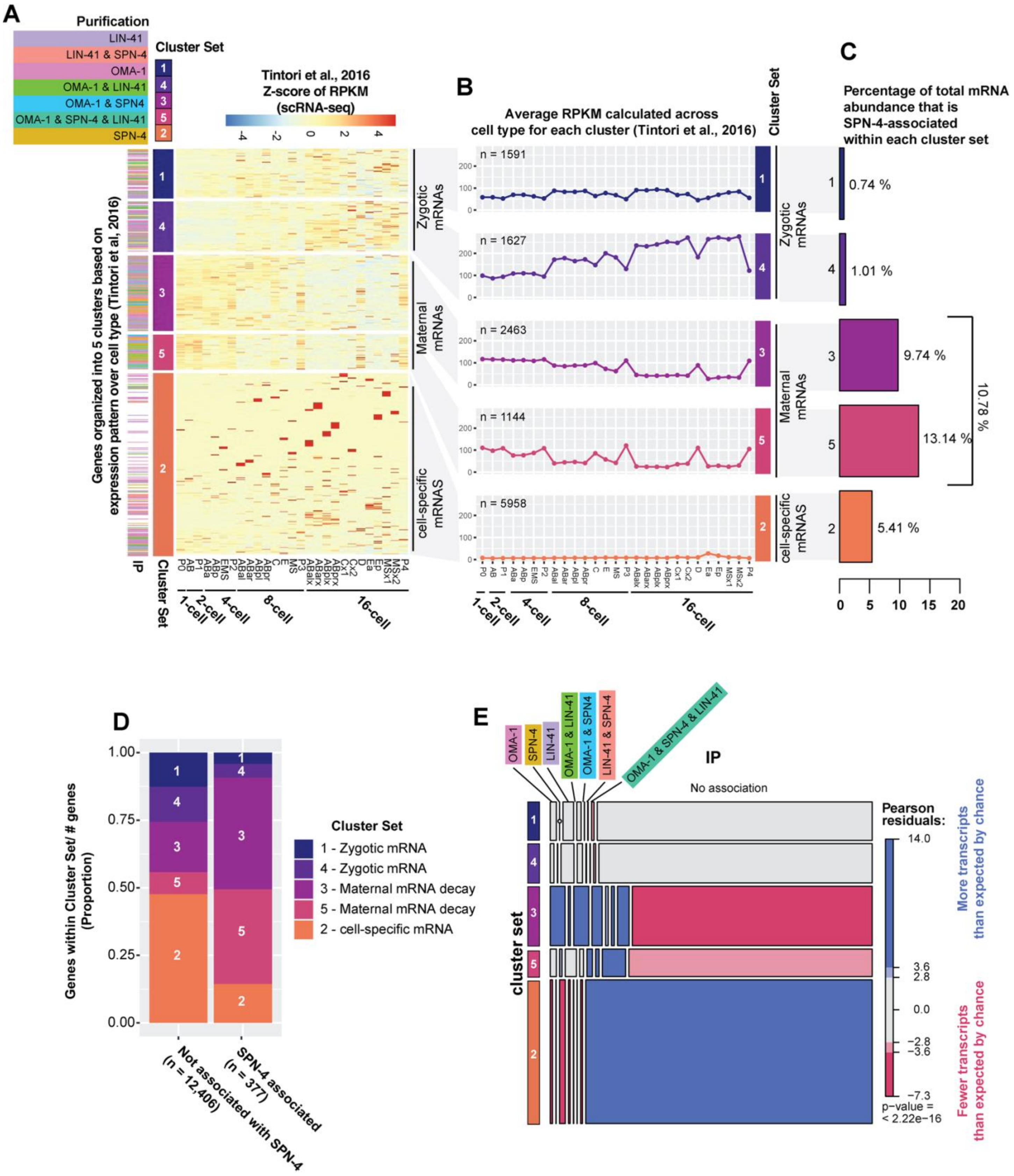

Development • Supplementary information

Fig. S6. Single-cell RNA-seqtr anscriptome profiles from the first 31 blastomeres. (Related to Fig. 2). (A) scRNA-seq transcriptome profiles from the first 31 embryonic blastomeres were reported in Tintori et al., (2016) (Tintori et al., 2016). That data is depicted here in heatmap form. Genes were filtered for total RPKM > 5 and variance > 10 to yield 14,776 genes. Heatmaps were generated with Canberra distance, complete clustering, row scaling and cutree = 5. SPN-4-, OMA-1- or LIN-41-associated mRNAs were annotated in the specified colors. Two clusters represented newly transcribed zygotic mRNAs (Cluster Sets 1 and 4), two represented maternal mRNAs that undergo decay (Cluster Sets 3 and 5), and one was comprised of zygotic mRNAs transcribed with cell-type specificity (Cluster Set 2). (B) Genes were clustered based on categories generated in hierarchical clustering in (A). Mean expression for genes across each cluster was calculated for each cell type and plotted as line plots showing mean (RPKM) over cell type. Cluster sets differentiate maternal mRNA from zygotic mRNA and those with cell-specific zygotic expression patterns. (C) The percentage of genes within each cluster set [generated in (A)] that have transcripts associated with SPN-4. (D) Categorical bar plots illustrating the representation of each Cluster Set (from A) among SPN-4-associated and -unassociated transcripts. (E) Contingency tables were generated from frequencies of SPN-4, OMA-1 and LIN-41 association and cluster identity. Contingency tables are shown plotted as Mosaic plots in which the area is proportional to frequencies. Mosaic plots are representations of contingency tables, which relate RNA-binding protein association to the membership in the various scRNA-seq clusters. Deviations between observed and expected numbers were quantified as Pearson residuals are shown, in which high residuals represent a larger observed membership than expected by chance and low residuals represent a smaller one. This illustrated that all RNA-binding protein-associated cohorts were over-represented in Cluster Set 3 and SPN-4-associated cohorts were over-represented in Cluster Set 5. Shading illustrates deviation from expected frequencies by chance according to log-linear modeling, with darker shading illustrating greater deviation from chance.

Development • Supplementary information

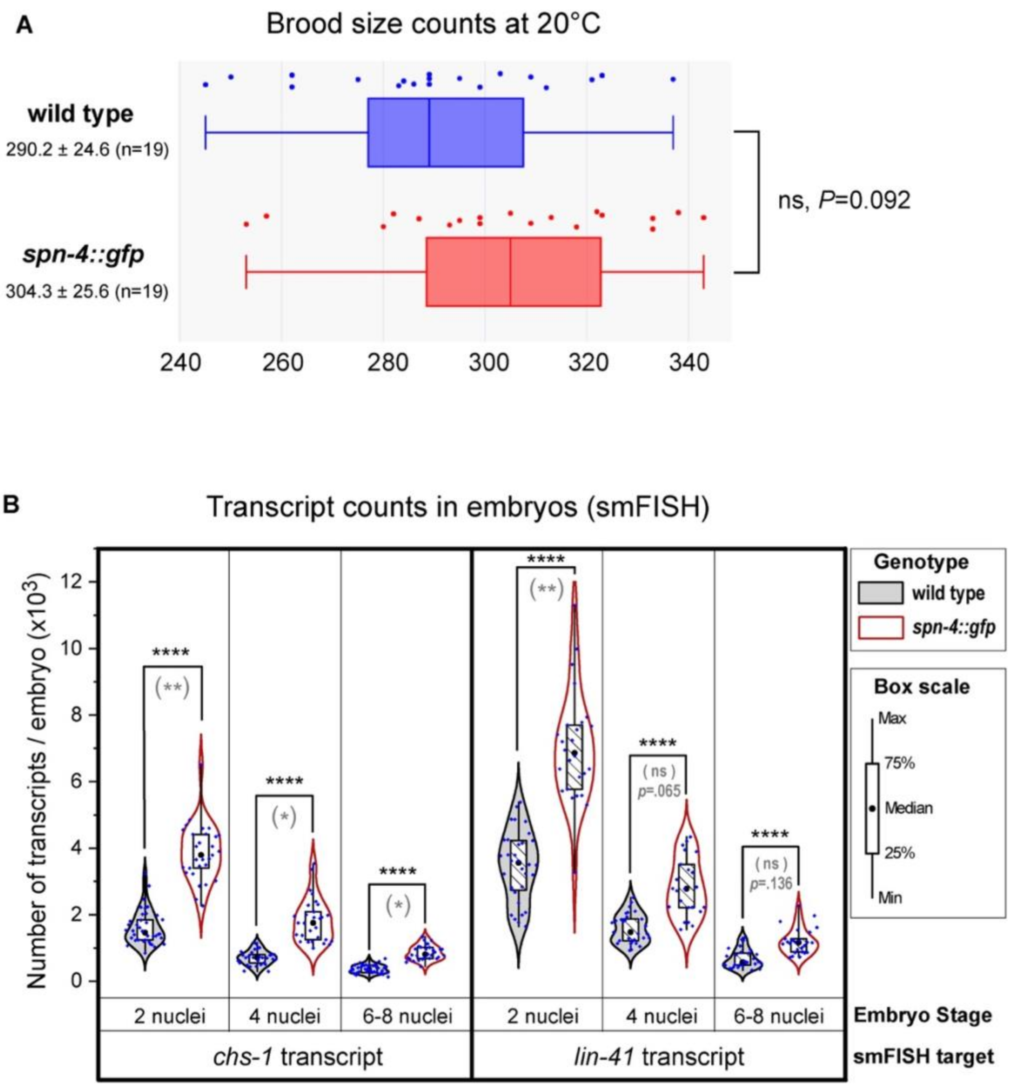

Fig. S7. SPN-4-associated transcripts are more abundant in *spn-4(tn1699[spn-4::gfp])* embryos, consistent with the idea that it is a weak hypomorph. (Related to Fig. 3). (A) Wild-type and DG4158 *spn-4(tn1699[spn-4::gfp])* animals have brood sizes that are not significantly different (unpaired Student's t-test). (B) *lin-41* and *chs-1* transcript numbers are increased in early DG4158 *spn-4(tn1699[spn-4::gfp])* embryos relative to the wild type. The graph shows violin and box plots with individual data points depicting the number of *chs-1* (left) or *lin-41* (right) transcripts in individual embryos at early developmental stages (2, 4 or 6-8 nuclei). These data were collectively analyzed for significance at each developmental stage. Black asterisks above a group-connecting bracket indicate the distribution is significantly different from the one observed for stage-matched wild-type embryos using Welch's one-way ANOVA, followed by a Games-Howell post-hoc test to account for unequal variances and multiple comparisons. Because some distributions failed the Shapiro-Wilk test for normality, we also report significance values derived from the non-parametric Kruskal-Wallis test, followed by Dunn's post-hoc test with Benjamini-Hochberg correction for multiple comparisons. Non-parametric significance results are gray and in parentheses. Two or three replicate experiments for each genotype and probe; total n-value >22 at each developmental stage. Significance values: **** P<0.0001, *** P< 0.001, ** P<0.01, * P<0.05, ns=not significant. Exact P and n values are reported in Table S14.

Development • Supplementary information

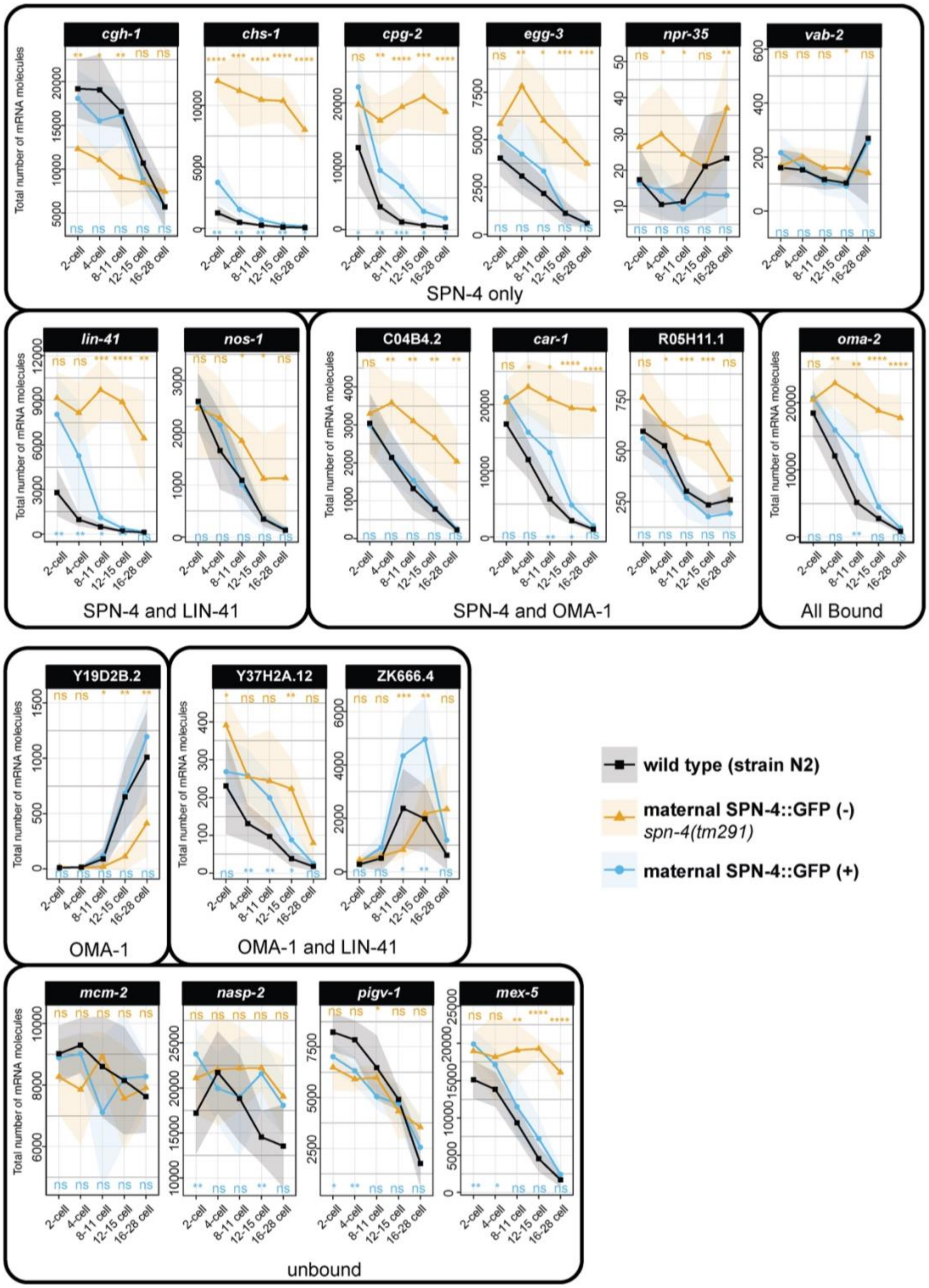

Fig S8. smFISH plots for selected transcripts. (Related to Fig. 3). The abundance of key transcripts in the wild type (strain N2) or without maternal *spn-4* activity as imaged by smFISH or smiFISH and quantified over five developmental stages. Transcripts are grouped according to their association with SPN-4, LIN-41 and/or OMA-1. Typically, 7 embryos (with a range of 4-12) per transcript, genotype and stage combination were collected across two to three replicates. Ribbons, standard deviation. Statistics = Welch's *t*-tests adjusted by Benjamini-Hochberg multiple test correction. ****: $P <= 0.0001$, ***: $P <= 0.001$, **: $P <= 0.01$, *: $P <= 0.05$ and ns: $P > 0.05$ (not significant). Exact *P* values are reported in Table S8.

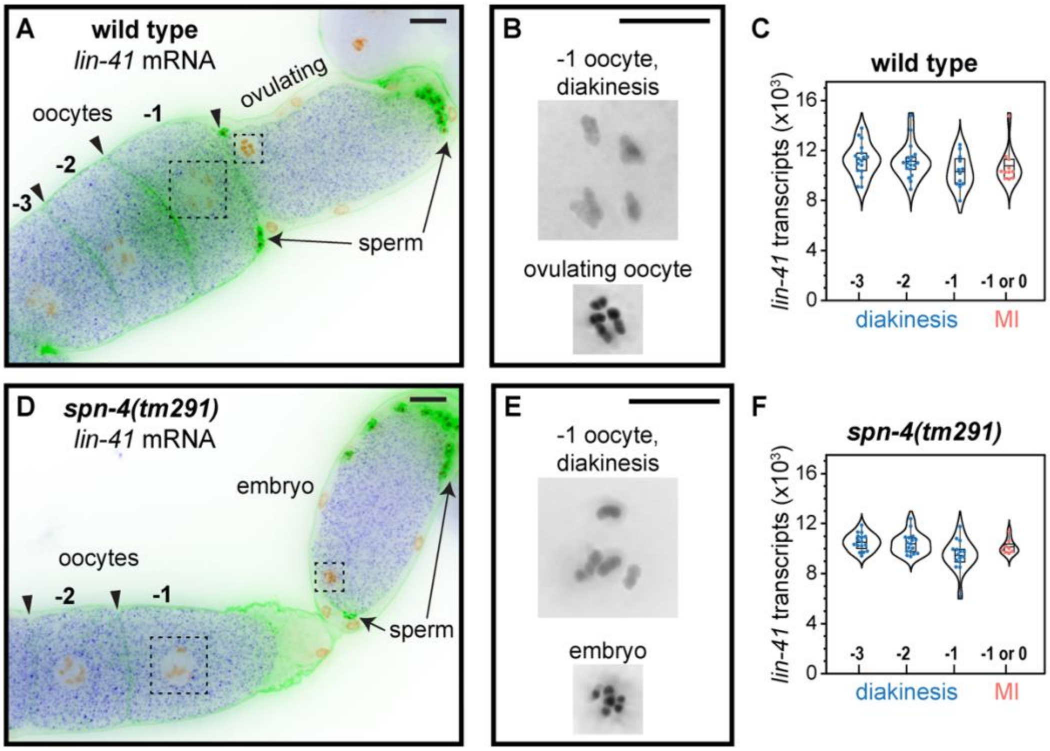

Fig. S9. *lin-41* transcripts numbers are stable in wild-type oocytes and are unaffected by SPN-4. (Related to Fig. 4). (A-F) smFISH analysis and quantification of *lin-41* transcripts (blue) in wild-type (A-C) and *spn-4(tm291)* (D-F) dissected gonads. WGA (green) was used to delimit oocyte boundaries (arrowheads) and to detect sperm. (B,E). The DNA channel (orange in (A,D)) is magnified to illustrate diakinesis and MI nuclei with a clustered arrangement of condensed chromosomes (B,E). Proximal oocytes (–1 to –3) are indicated. Bars, 10 μ m. Raw data, exact *P* values and n values are shown in Table S9.

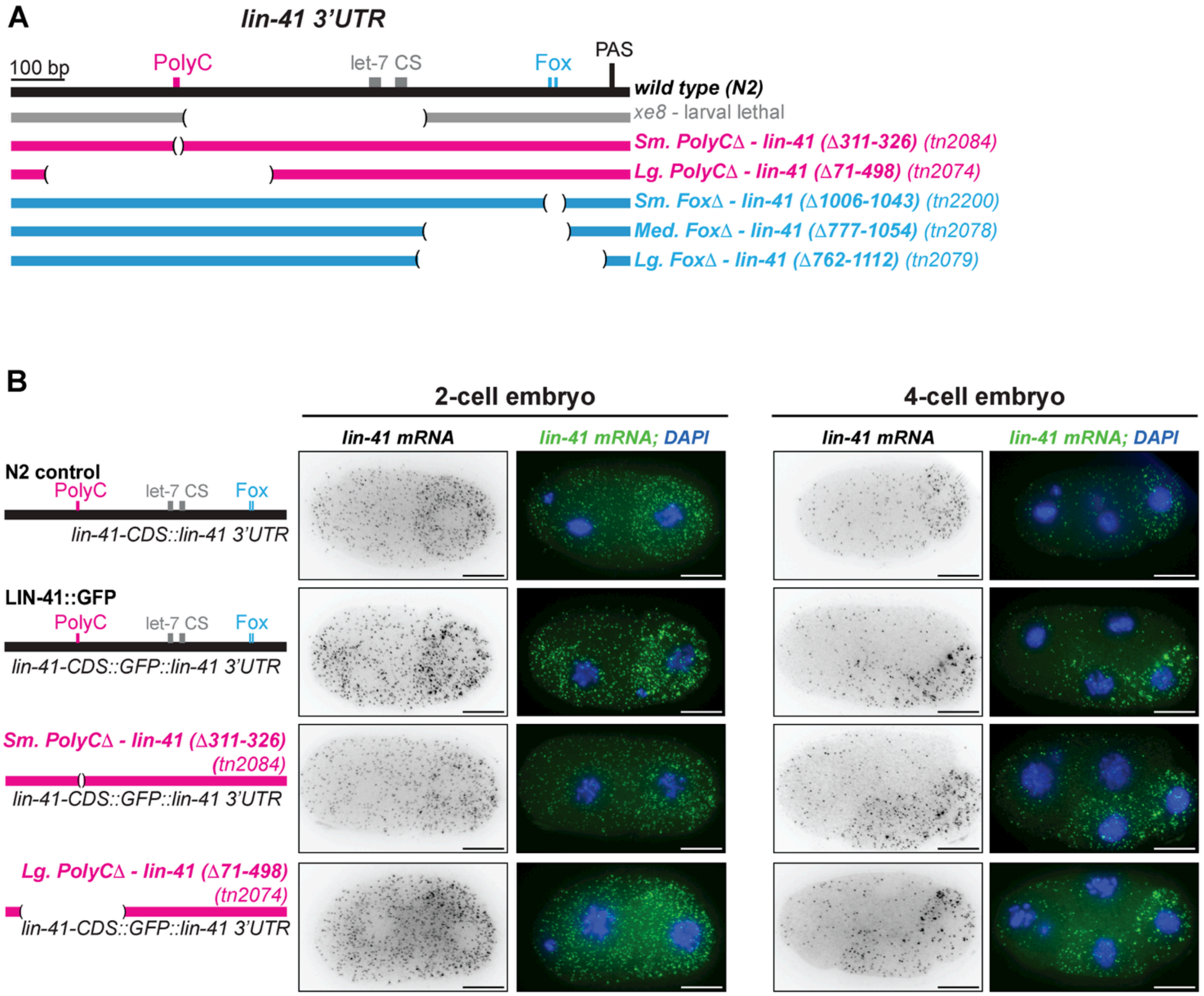

Fig. S10. The polyC RNA sequence motif in the *lin-41* 3'UTR is not required for maternal mRNA clearance. (Related to Fig. 6). (A) The PolyC sequence within the *lin-41* 3'UTR was disrupted as either a small (Δ311-326) or large (Δ71-498) deletion in the *lin-41(tn1541[gfp::lin-41])* genetic background. (B) The impact of the polyC-motif deletions on *lin-41* mRNA abundance, compared to the *lin-41(tn1541[gfp::lin-41])* or wild type (N2) control, was assessed by smFISH. Images are representative of 9- 31 embryos of each condition combination assayed over 2-4 replicates. The wild-type images in B are also displayed in Fig. 6B. For purposes of direct comparison, these wild-type images were reprocessed in parallel with the polyC image shown here. Exact *P* and n values are reported in Table S10. Bars,10 μm.

Development • Supplementary information

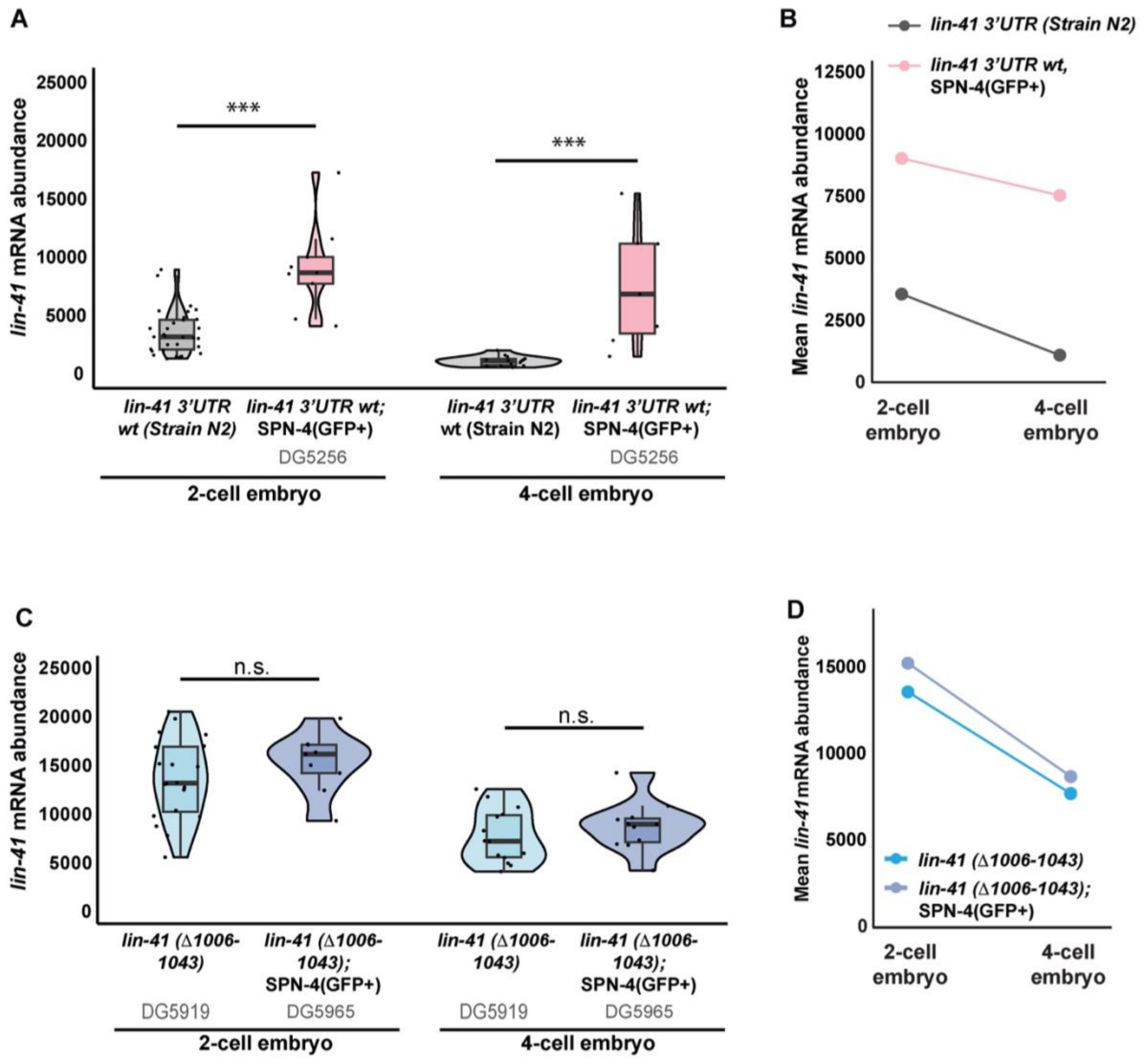

Fig. S11. SPN-4::GFP, which is a hypomorph, does not synergize with the *lin-41* Rbfox-motif deletion. (Related to Fig. 7). (A) Comparison of *lin-41* mRNA levels in embryos produced by the wild type (strain N2) and embryos from strain DG5256 *spn- 4(tm291)/tmC3[egl-9(tmIs1230) spn-4(tn2056[spn-4::gfp::3xflag])]* that have a maternal *spn-4::gfp(+)* genotype. Embryos that express SPN-4::GFP exhibit elevated levels of *lin-41* transcripts compared to the wild type. (B) Same dataset as in (A) but with mean *lin-41* mRNA abundance calculated and plotted across the two cell stages. (C) A comparison of *lin-41* mRNA levels from embryos produced by strain DG5919 *lin-41(tn2238Δ*1006-1043*)*, which lacks the Rbfox-motif sequence in its 3'UTR, and embryos produced by strain DG5965 *lin-41(tn2238Δ* 1006-1043*); spn-4(tm291)/tmC3[egl-9(tmIs1230) spn-4(tn2056[spn-4::gfp::3xflag])]* that have a maternal *spn-4::gfp(+)* genotype. (D) Same data as (C) but calculating mean *lin-41* mRNA abundance and comparing across cell stages. This result supports the conclusion that SPN-4 functions mainly through the Rbfox motif to regulate *lin-41* maternal mRNA clearance. For all assays: n > 8 for each condition. Statistical tests were performed for each cell stage using ANOVA analysis, and pairwise differences were assessed using the post-hoc Tukey's Honest Significant Differences analysis to account for multiple testing. Raw data and statistics are in Table S11. *** *P*-adj < 0.001; ** *P*-adj < 0.01 ; * *P*-adj < 0.05; ns = not significant.

Development • Supplementary information

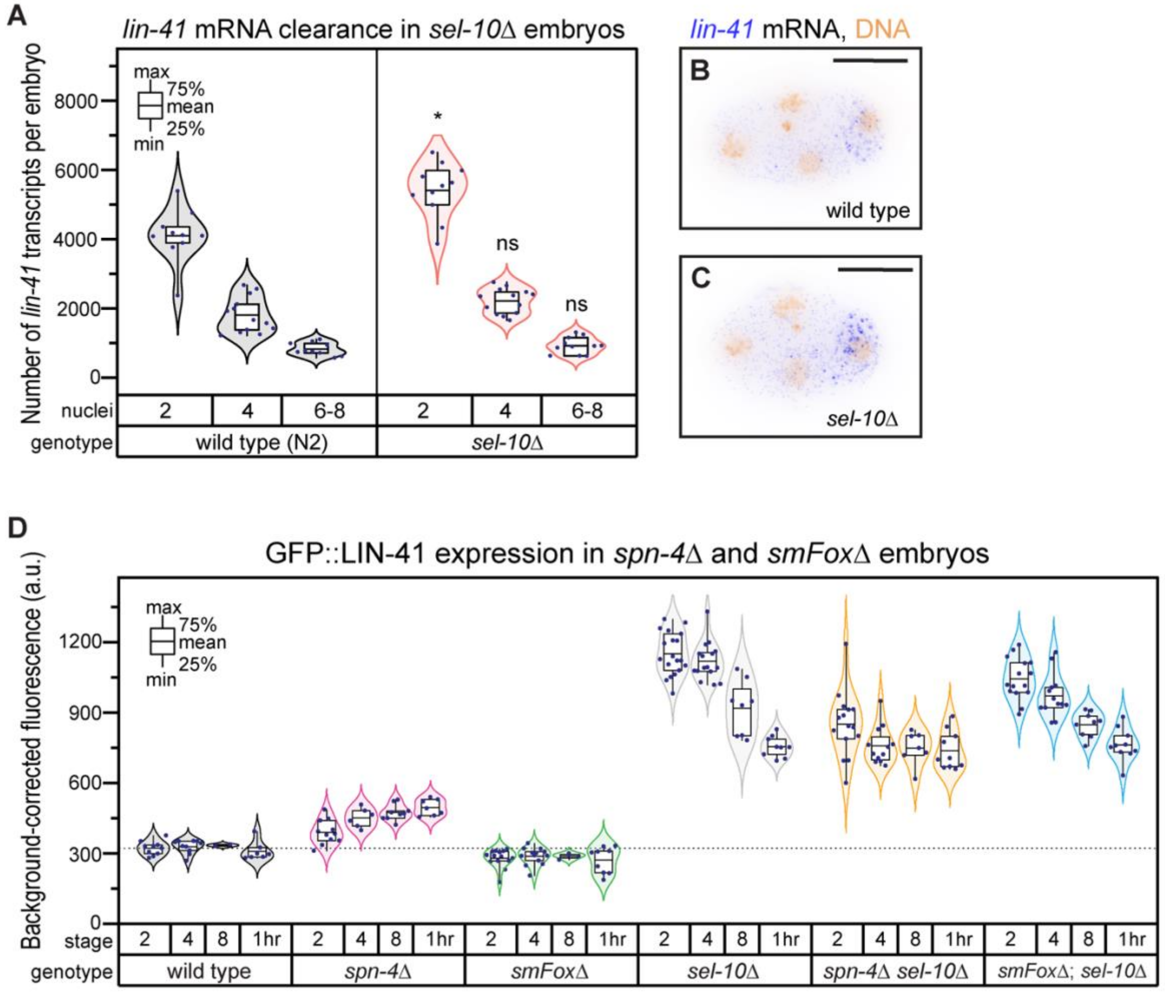

Fig. S12. A *sel-10* null mutation does not affect clearance of *lin-41* mRNA and analysis of GFP::LIN-41 levels in *sel-10* and *smFoxΔ* mutant backgrounds. (Related to Fig. 9). (A-C) smFISH data for *lin-41* in the wild type and *sel-10(ok1632)* null mutant (*sel-10Δ*) embryos. Quantitative (A) and representative smFISH (B,C) data. Bars, 20 μm. Note *lin-41* transcript levels are slightly elevated in *sel-10Δ* mutants at the 2-cell stage (*P*=0.012, asterisk, using Welch's one-way ANOVA with a Games-Howell post-hoc test), however, *lin-41* transcript levels are not significantly (ns) different from the wild type at the 4- and 6-8-cell stages. See Table S15 for exact *P* and n values. (D) Quantitative analysis of background-corrected GFP::LIN-41 fluorescence levels in 2-cell, 4-cell, 8-cell and "1hr"-embryos (1hr-embryos were examined 1 hr after the 2-cell stage, which corresponds approximately to the 24-cell stage in the wild type). The dotted line indicates the average fluorescence levels of all wild-type embryos, which is attributed to autofluorescence. Genotypes: wild type–DG3913 *lin-41(tn1541[gfp::s-tag::lin-41])* I, *spn-4Δ–lin-41(tn1541[gfp::s-tag::lin-41])* I; *spn-4(tm291)* V from DG4517, *smFoxΔ*–DG5779 *lin-41(tn1541[gfp::s-tag::lin-41] tn2200)* I, *sel-10Δ*–DG4310 *lin-41(tn1541[gfp::s-tag::lin-41])* I; *sel-10(ok1632)* V, *spn-4Δ sel-10Δ–lin-41(tn1541[gfp::s-tag::lin-41]* I; *spn-4(tm291) sel-10(ok1632)* V from DG4549, *smFoxΔ sel-10Δ*–DG6272 *lin-41(tn1541[gfp::s-tag::lin-41]) tn2200)* I; *sel-10(ok1632)* V. GFP::LIN-41 expression data and n values are in Table S13.

Development • Supplementary information

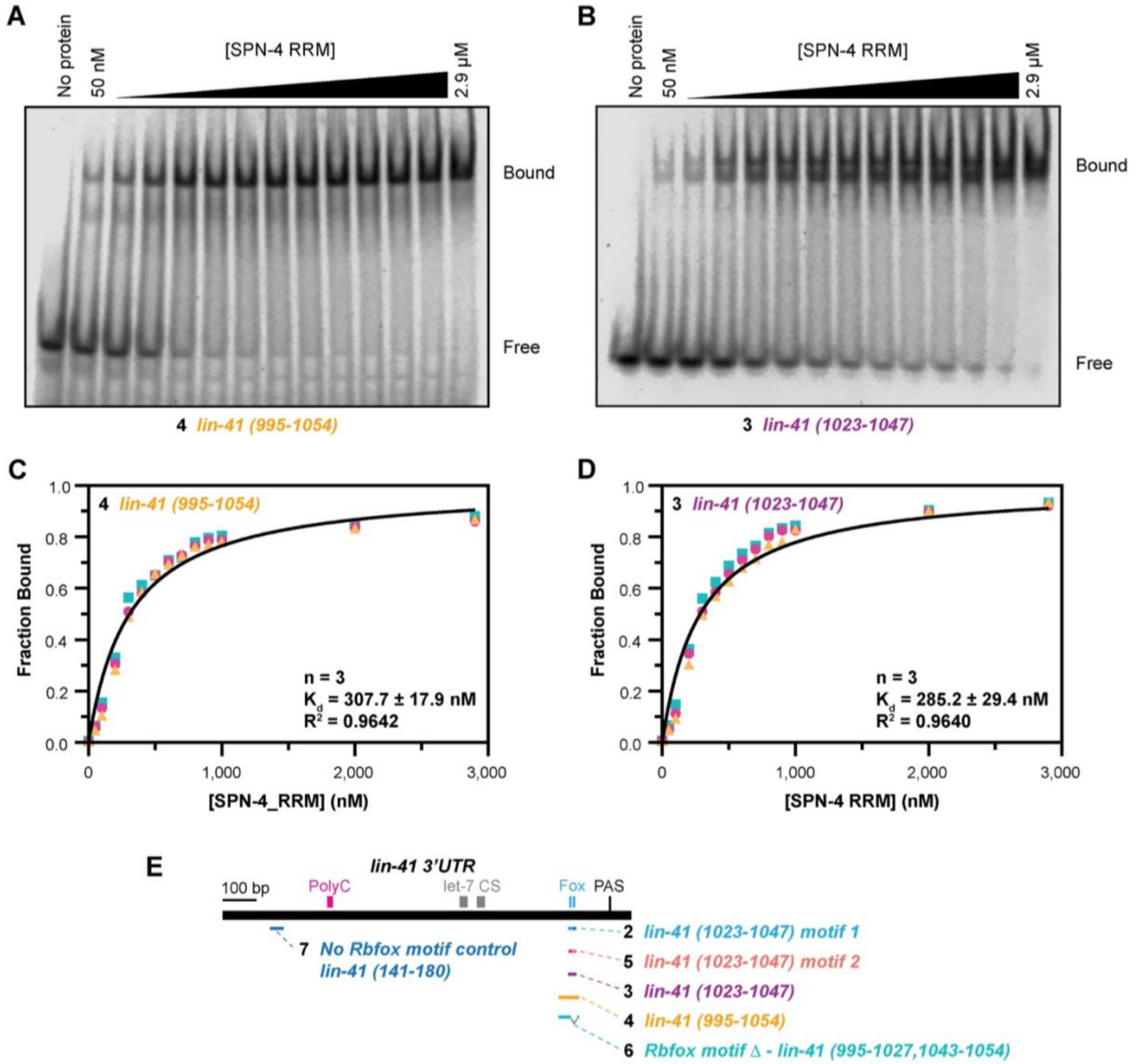

Fig. S13. *In vitro* binding assays for the 60-nt and 25-nt RNA probes that contain the *lin-41* Rbfox motif. (Related to Fig. 10). (A,B) Representative *in vitro* binding EMSA assays for the interaction of the SPN-4 RRM with the *lin-41(995-1054)* RNA probe (A) or the *lin-41(1023-1047)* RNA probe (B) run on native 8% polyacrylamide gels. The binding data in A is also shown in Fig. 10A and is shown here for purposes of comparison. (C,D) Lineplot curves plotting the mean fraction bound over SPN-4 RRM concentration. Calculated Kd values are shown. n=3 replicates. (E) A map of the *lin-41* 3'UTR showing the position of the probes. The sequences of the probes are shown in Fig. 10D. To assess whether the SPN-4 RRM might bind cooperatively, we calculated the Hill coefficient (h) using the equation log(Y/1-Y)=hlog[C]-logKd; where Y is the fraction of bound RNA and [C] is the concentration of the SPN-4 RRM. For binding to the 60-*nt lin-41(995-1054)* RNA probe, h=1.03, indicating that the binding is not cooperative.

Development • Supplementary information

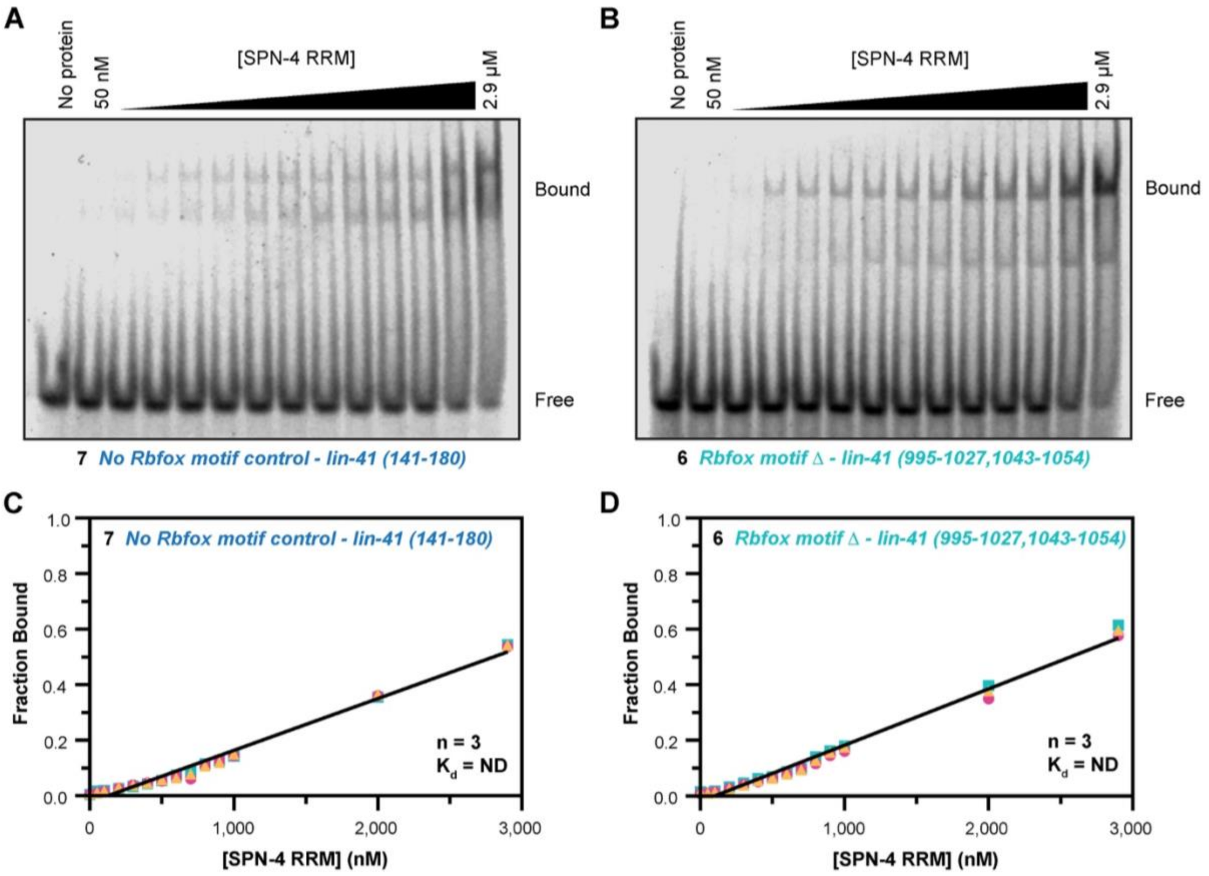

Fig. S14. *In vitro* binding assays for the 40-nt and 43-nt RNA probes that do not contain the *lin-41* Rbfox motif. (Related to Fig. 10). (A,B) Representative *in vitro* binding EMSA assays for an interaction between the SPN-4 RRM and the *lin-41(141-180)* control RNA probe (A) or the *lin-41(995-1027,1043-1054)* control RNA probe (B) that lacks the Rbfox motif sequence. Assays were run on native 8% polyacrylamide gels. The binding data in B is from Fig. 10B and is shown here for purposes of comparison. (C,D) Lineplot curves plotting the mean fraction bound over SPN-4 RRM concentration. The SPN-4 RRM exhibits non-specific and non-saturable binding to these control probes. n=3 replicates.

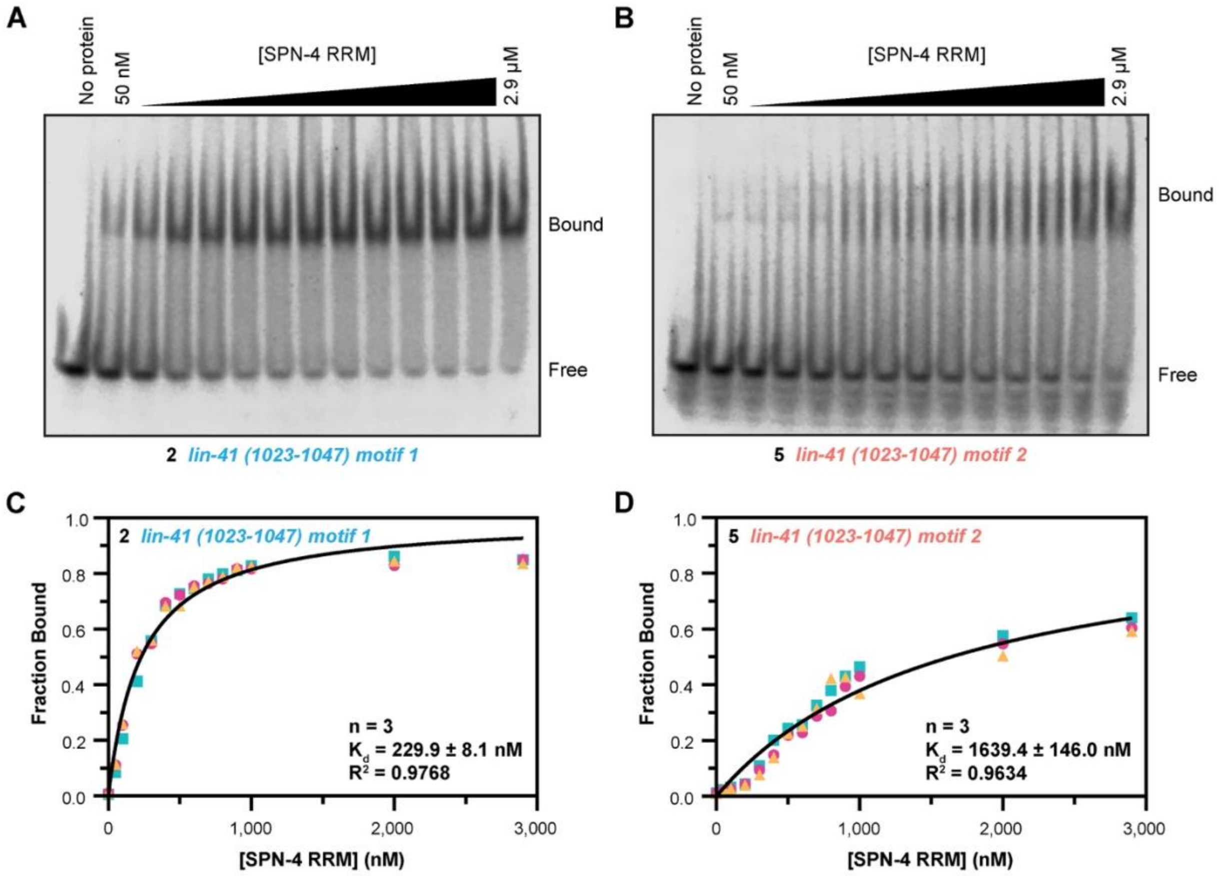

Fig. S15. SPN-4 preferentially binds RNA containing the Rbfox consensus sequence in motif 1. (Related to Fig. 10). (A, B) Representative *in vitro* binding EMSA assays for an interaction between the SPN-4 RRM and the *lin-41(1023-1047)* motif 1-containing RNA probe (A) or the *lin-41(1023-1047)* motif 2-containing RNA probe. Assays were run on native 8% polyacrylamide gels. (C,D) Lineplot curves plotting the mean fraction bound over SPN-4 RRM concentration. Calculated Kd values are shown. n=3 replicates.

Development • Supplementary information

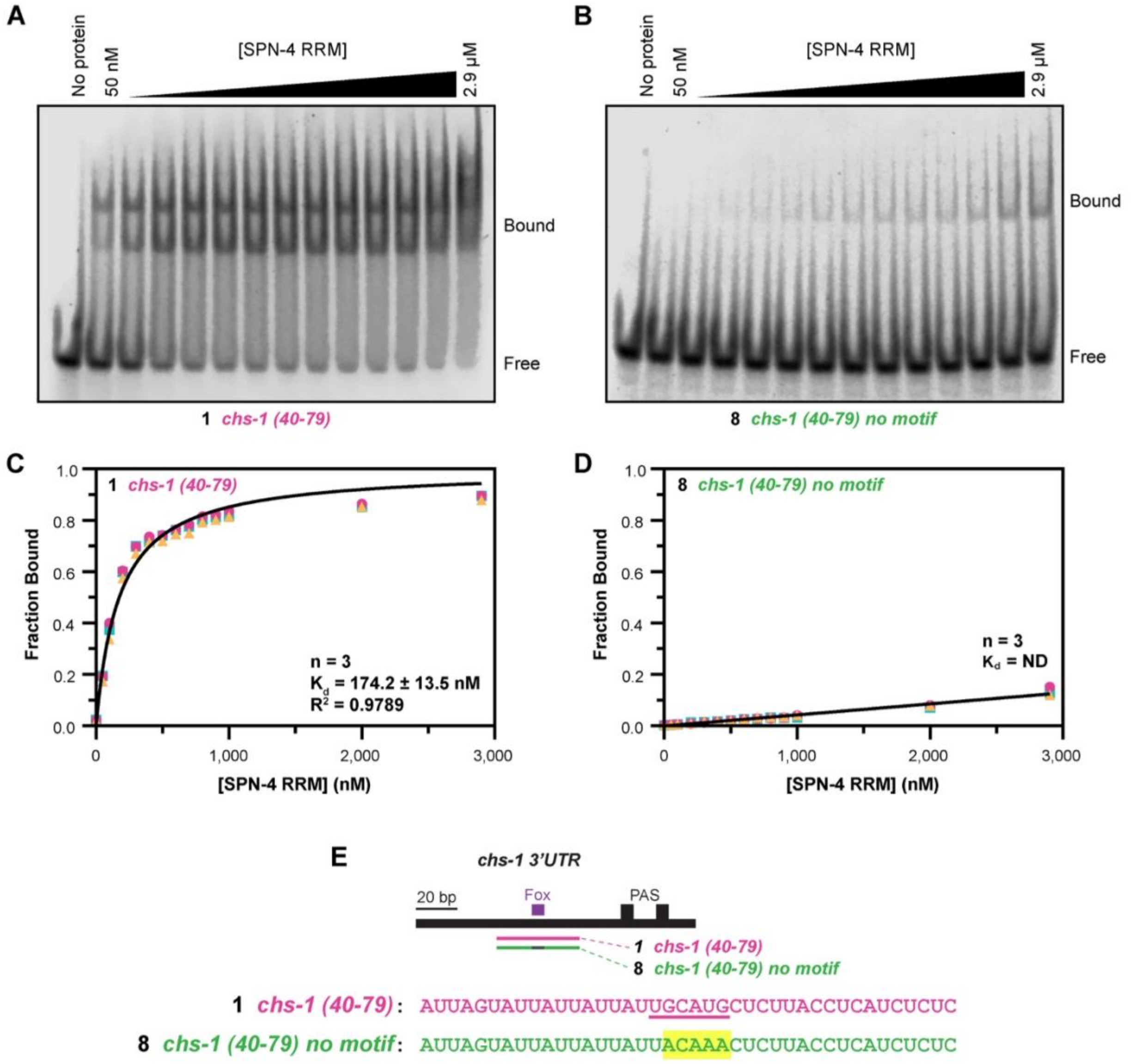

Fig. S16. The SPN-4 RRM binds the *chs-1* 3'UTR. (Related to Fig. 10). (A,B) Representative *in vitro* binding EMSA assays for the interaction of the SPN-4 RRM with a 40-nt chs-1(40-79) RNA probe that contains the Rbfox motif (A) or a control probe with base substitutions (B). Assays were run on native 8% polyacrylamide gels. (C,D) Lineplot curves plotting the mean fraction bound over SPN-4 RRM concentration. Calculated Kd values are shown. n=3 replicates. (E) Map of the chs-1 3'UTR and the sequence of the chs-1 probes. For binding of the SPN-4 RRM to the chs-1 RNA probe, we observed a doublet of two bound species. To ascertain whether the SPN-4 RRM might bind cooperatively to the chs-1 probe, we calculated the Hill coefficient (h) using the equation log(Y/1-Y)=hlog[C]-logKd; where Y is the fraction of bound RNA and [C] is the concentration of the SPN-4 RRM. For binding to the 40-nt chs-1(40-79) RNA probe, h=0.79, indicating that the binding is not cooperative. The basis for the formation of a doublet is not known.

Development • Supplementary information

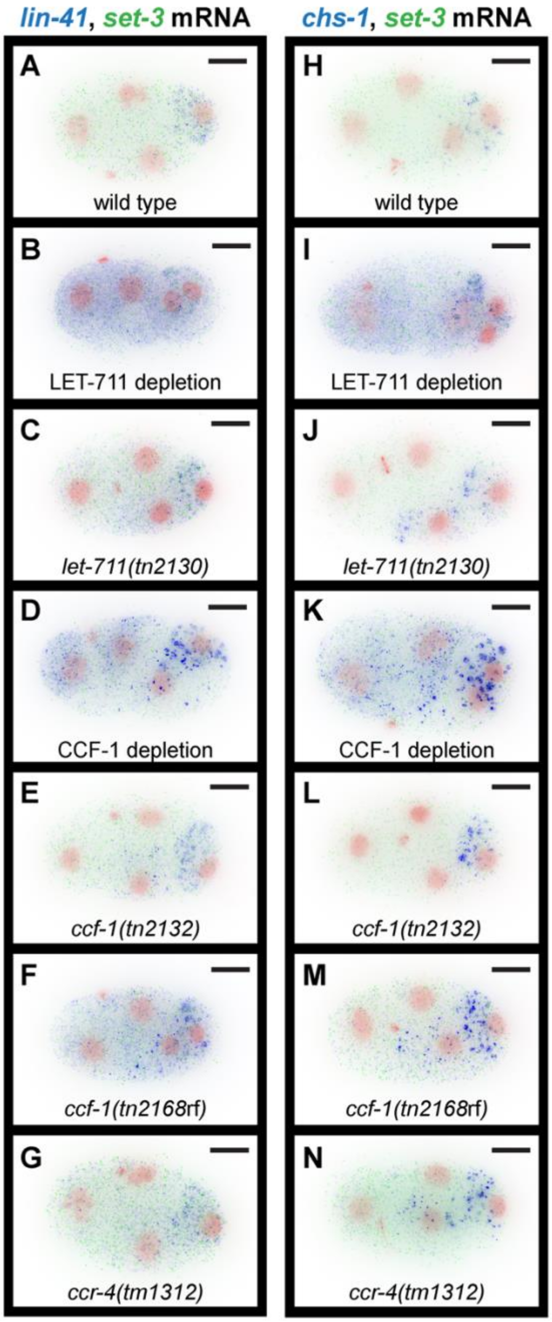

Fig. S17. The CCR-4-NOT complex mediates *lin-41* and *chs-1* mRNA clearance. (Related to Fig. 12G-N). Flourescence micrographs showing representative two-colour smFISH data for *lin-41* (blue in panels A-G), or *chs-1* (blue in panels H-N) and *set-3* (green) transcripts in wild-type, LET-711 depletion, *let-711(tn2130)*, CCF-1 depletion*, ccf-1(tn2132)*, *ccf-1(tn2168*rf*)* or *ccr-4(tm1312)* embryos with 4 nuclei (DNA, salmon). Two or three replicate experiments for each genotype; total n values > 21. Panels A,B,D,F,H,I,K and M are from Fig. 12G-N and are shown here for purposes of comparison. Bars, 10 μm.

Development • Supplementary information

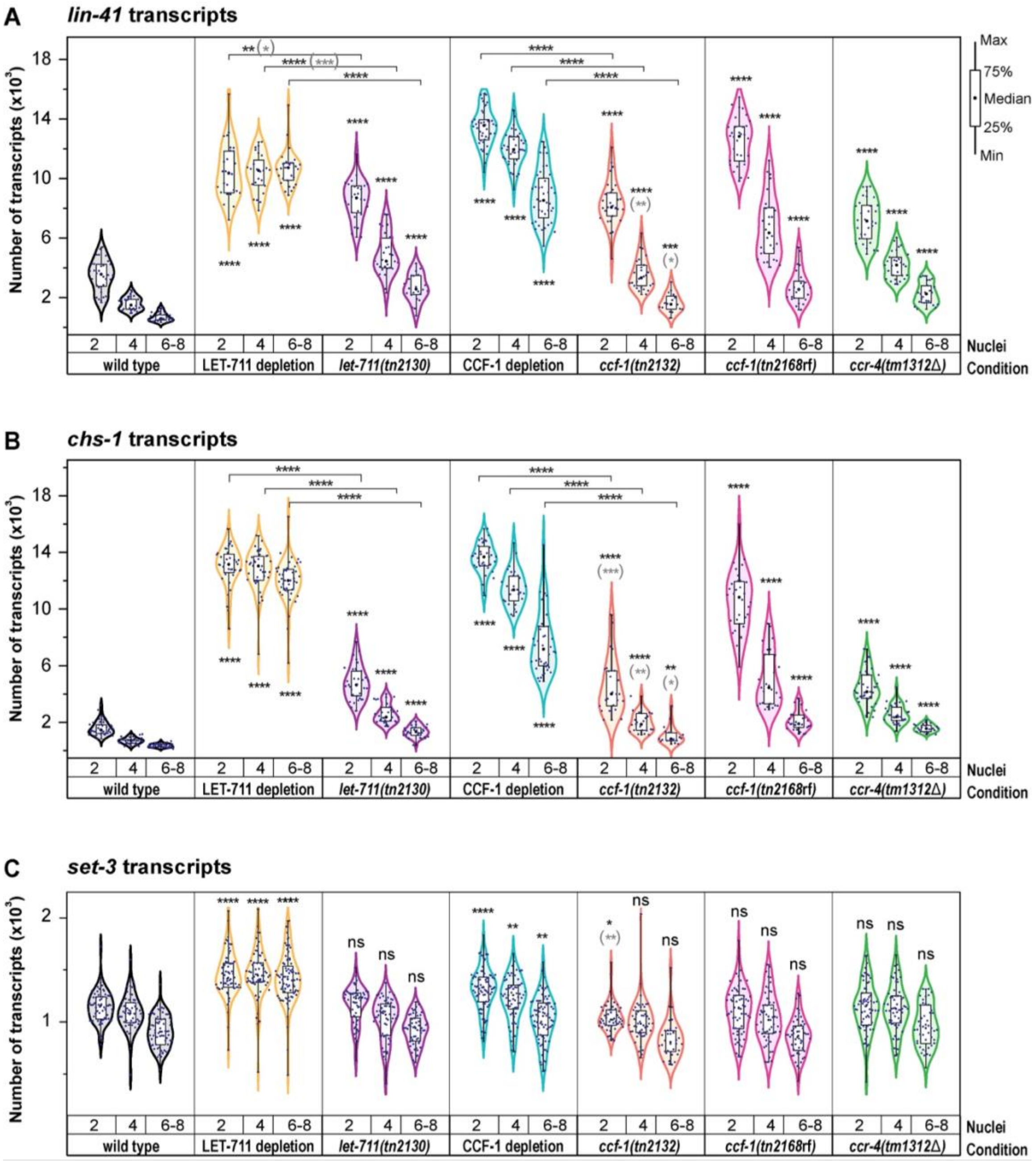

Fig. S18. ***lin-41*****,** ***chs-1*** **and** ***set-3*** **smFISH data related to the CCR-4-NOT complex.** (Related to Fig. 12). (A,B) *lin-41* and *chs-1* transcript numbers increased in early embryos when the CCR-4-NOT complex subunits (LET-711, CCF-1 and CCR-4) were mutated or depleted, with large and more durable effects (up to 32-fold) occurring under the LET-711AID and CCF-1AID protein depletion conditions that cause embryonic lethality. (C) *set-3* transcript numbers were slightly increased by LET- 711AID and CCF-1AID protein depletion (up to 1.6-fold). All graphs show violin and box plots with individual data points depicting the number of *lin-41* (A), *chs-1* (B) and *set-3* (C) transcripts in individual embryos at each developmental stage (2, 4 or 6-8 nuclei) and condition (genetic background or auxin-dependent protein depletion). Small black asterisks (not above brackets but located below or above a transcript count distribution) indicate the distribution was significantly different from wild-type (far left panels in A-C). We also compared the LET-711 AID and CCF-1AID depletions

Development • Supplementary information

to embryos expressing the same AID-tagged fusion protein under non-depleting conditions (no TIR1 driver or auxin analog). These control embryos had the following genotypes: *let-711(tn2130[let-711::gfp::aid*::3xflag])* or *ccf-1(tn2132[ccf-1::gfp::aid*::3xflag])*. Large black asterisks above a connecting bracket indicate that the transcript counts observed after AID protein depletion were significantly different from controls lacking the TIR1 driver and auxin analog. Significance values in black were obtained using Welch's one-way ANOVA, followed by a Games-Howell post-hoc test to account for unequal variances and multiple comparisons. Because some distributions failed the Shapiro-Wilk test for normality, we report significance values derived from the non-parametric Kruskal-Wallis test, followed by Dunn's post-hoc test with Benjamini-Hochberg correction for multiple comparisons. Gray asterisks representing these non-parametric significance results are in parentheses; they are only shown when the significance value differs from the Welch's one-way ANOVA result. Significance values: ****P<0.0001, ***P< 0.001, ** P<0.01, * P<0.05, ns=not significant. Two or three replicate experiments for each genotype and two-color probe combination (e.g., *set-3* + *lin-41* transcript detection). Total n values greater than or equal to 16 at each developmental stage in the different *lin-41* and *chs-1* experiments. Total n value >33 at each developmental stage for set-3. Exact P and n values are reported in Table S14.

Development • Supplementary information

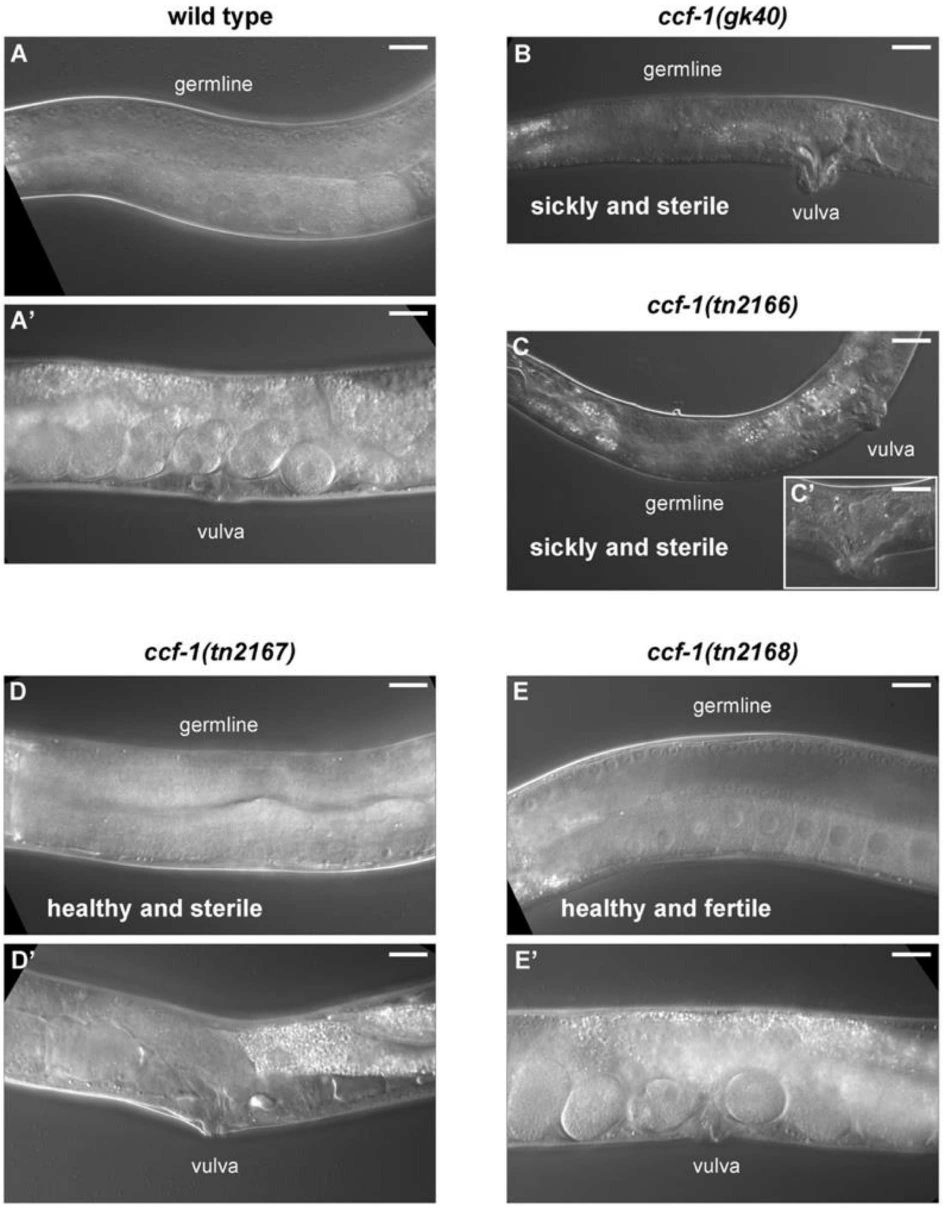

Fig. S19. A *ccf-1* allelic series. (Related to Fig. 11). (A-E') DIC images of adult hermaphrodites of the wild type (A,A') and *ccf-1* mutants (B-E'), which form an allelic series. *ccf-1(gk40)* is a null allele (WormBase; Sternberg et al., 2024). The *ccf-1(tn2166-tn2168)* mutant alleles were generated by CRISPR-Cas9 genome editing (see Materials and Methods). *ccf-1(tn2168)* is fertile, whereas the other *ccf-1* alleles exhibit sterility. Bars, 20 μm.

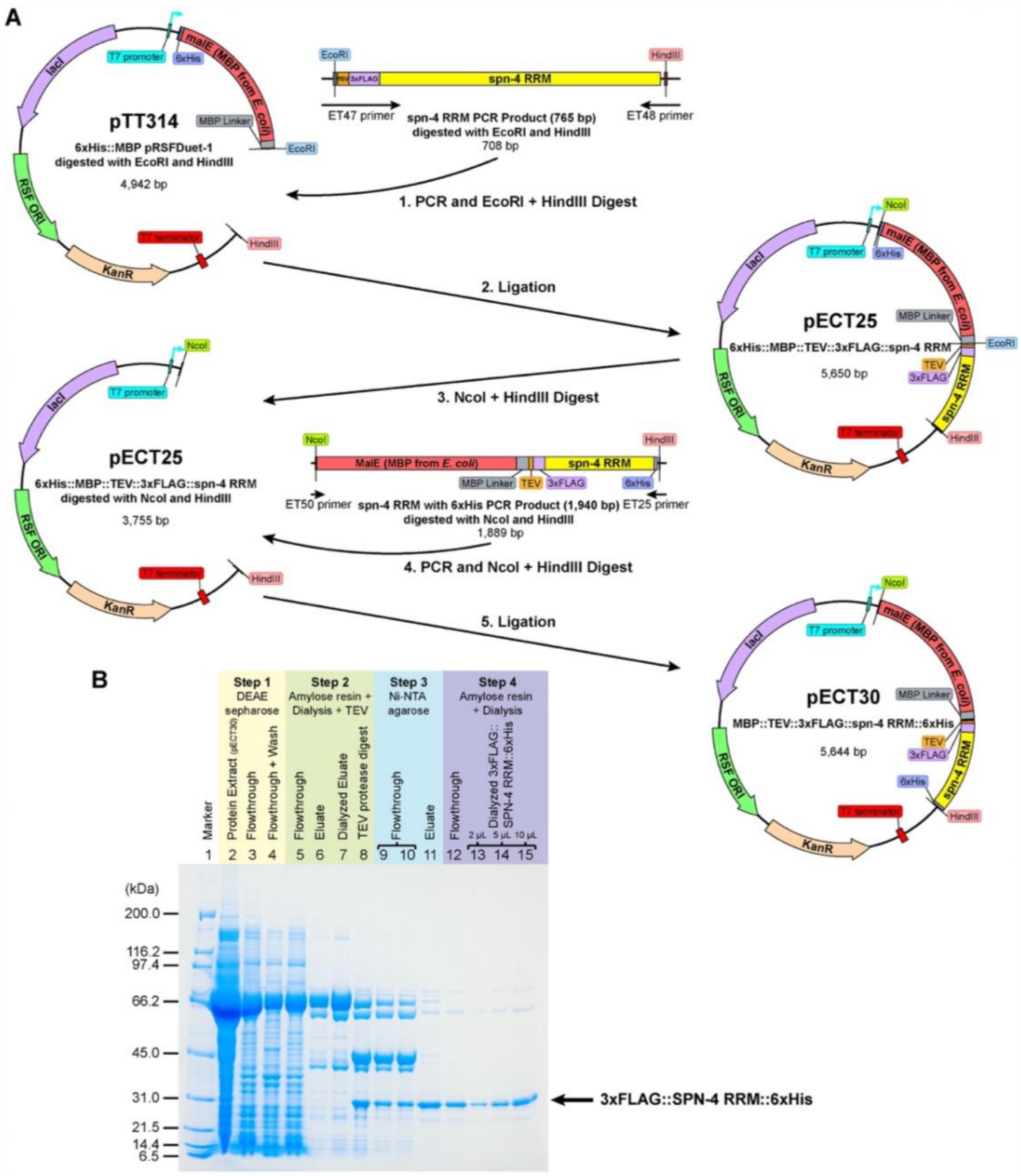

Fig. S20. Overview of SPN-4 RRM expression plasmid construction and purification. (Related to Materials and Methods and Figs 10 and S13-16). (A) Schematic of cloning methods used to generate the SPN-4 RRM expression plasmid, pECT30. (B) A colloidal Coomassie-stained protein 4-12% Bis-Tris NuPAGE gel showing steps used to purify the SPN-4 RRM. All the RNA-binding experiments used the SPN-4 RRM shown in lanes 13-15. The gel is representative of two biological replicates.

Table S1. LIN-41-, OMA-1 and SPN-4-associated transcripts identified by immunopurification of RNA-binding proteins and RNA sequencing. (Related to Fig. 1).

Available for download at
https://journals.biologists.com/dev/article-lookup/doi/10.1242/dev.205295#supplementary-data

Development • Supplementary information

Table S2. *C. elegans* strains used for this study

Strain	Genotype
N2	Wild type, Bristol isolate
EV960	*ccr-4(tm1312)* IV/*nT1[qIs51]* IV;V
FX30234	*tmC9[F36H1.2(tmIs1221)]* IV
GS6156	*sel-10(ok1632)* V
JK560	*fog-1(q253*ts*)* I
JK5996	*puf-11(q971)* IV
JK6321	*puf-11(q971) puf-3(q966)* IV/*nT1[qIs51]* IV; V
DG2566	*fog-1(q253*ts*)* I; *oma-1(zu405te33)* IV; *tnIs17[pCS410 oma-1p::oma-1::s-tag::tev::gfp, unc-119(+)]*
DG2581	*spe-9(hc88*ts*)* I; *oma-1(zu405te33)* IV; *tnIs17[pCS410 oma-1p::oma-1::s-tag::tev::gfp, unc-119(+)]*
DG3913	*lin-41(tn1541[gfp::s-tag::lin-41])* I
DG3923	*fog-1(q253*ts*) lin-41(tn1541[gfp::tev::s-tag::lin-41])* I
DG4158	*spn-4(tn1699[spn-4::gfp::3xflag])* V
DG4310	*lin-41(tn1541[gfp::s-tag::lin-41])* I; *sel-10(ok1632)* V
DG4398	*fog-1(q253*ts*)* I; *spn-4(tn1699[spn-4::gfp::tev::3xflag])* V
DG4400	*spe-9(hc88*ts*)* I; *spn-4(tn1699[spn-4::gfp::tev::3xflag])* V
DG4485	*spe-9(hc88*ts*) lin-41(tn1541[gfp::tev::s-tag::lin-41])* I
DG4517	*lin-41(tn1541[gfp::s-tag::lin-41])* I; *spn-4(tm291)/tmC3[tmIs1230]* V
DG4549	*lin-41(tn1541[gfp::s-tag::lin-41])* I; *spn-4(tm291) sel-10(ok1632)* V/*nT1[qIs51]* IV;V
DG5216	*spn-4(tm291)/tmC3[egl-9(tmIs1230)]* V
DG5256	*spn-4(tm291)/tmC3[egl-9(tmIs1230) spn-4(tn2056[spn-4::gfp::3xflag])]* V
DG5324	*puf-11(q971) tmC9[F36H1.2(tmIs1221)]* IV
DG5329	*lin-41(tn1541[gfp::s-tag::lin-41] tn2074[lgPolyCΔ in 3'UTR]* I
DG5364	*puf-11(q971) puf-3(q966)/puf-11(q971) tmC9[F36H1.2(tmIs1221)]* IV; *spn-4(tn1699[spn-4::gfp::3xflag])* V
DG5365	*puf-11(q971) puf-3(q966)/puf-11(q971) tmC9[F36H1.2(tmIs1221)]* IV
DG5398	*lin-41(tn1541[gfp::s-tag::lin-41] tn2078[medFoxΔ in 3'UTR]* I
DG5399	*lin-41(tn1541[gfp::s-tag::lin-41] tn2079[lgFoxΔ in 3'UTR]* I
DG5410	*lin-41(tn1541[gfp::s-tag::lin-41] tn2084[smPolyCΔ in 3'UTR]* I
DG5440	*puf-11(q971) puf-3(q966)/puf-11(q971) tmC9[F36H1.2(tmIs1221)]* IV; *spn-4(tm291)/tmC3[egl-9(tmIs1230)]* V
DG5444	*let-711(tn2102)/+* III; *puf-11(q971) puf-3(q966)* IV [a]
DG5454	*puf-11(q971) puf-3(q966)/puf-11(q971) tmC9[F36H1.2(tmIs1221)]* IV; *+/tmC3[egl-9(tmIs1230)]* V
DG5458	*let-711(tn2109)/+* III; *puf-11(q971) puf-3(q966)* IV [a]
DG5459	*ccf-1(tn2110)/+* III; *puf-11(q971) puf-3(q966)* IV [a]
DG5464	*puf-11(q971) puf-3(q966)/puf-11(q971) tmC9[F36H1.2(tmIs1221)]* IV; *spn-4(or191*ts*)/tmC3[egl-9(tmIs1230)]* V
DG5487	*let-711(tn2087)/+* III; *puf-11(q971) puf-3(q966)* IV [b]
DG5491	*let-711(tn2105)/+* III; *puf-11(q971) puf-3(q966)* IV [c]
DG5492	*let-711(tn2106)/+* III; *puf-11(q971) puf-3(q966)* IV [c]
DG5493	*let-711(tn2108)/+* III; *puf-11(q971) puf-3(q966)* IV [c]
DG5517	*puf-11(q971) puf-3(q966)* IV; *spn-4(tn2091)/+* V [c]
DG5530	*let-711(tn2088)* III; *puf-11(q971) puf-3(q966)* IV [b]
DG5533	*let-711(tn2082)/+* III; *puf-11(q971) puf-3(q966)* IV [d]

Development • Supplementary information

DG5538	*puf-11(q971) puf-3(q966)/puf-11(q971) tmC9[F36H1.2(tmIs1221)]* IV; *spn-4(tn2091)/tmC3[egl-9(tmIs1230)]* V
DG5632	*let-711(tn2130[let-711::gfp::aid::3xflag])* III
DG5634	*ccf-1(tn2132[ccf-1::gfp::aid::3xflag])* III
DG5671	*+/hT2[umnIs60(myo-2p::mKate2)]* I; *+/hT2[bli-4(e937)]* III; *puf-11(q971) puf-3(q966)/puf-11(q971) tmC9[tmIs1221]* IV
DG5672	*+/hT2[umnIs60(myo-2p::mKate2)]* I; *let-711(tn2135)/hT2[bli-4(e937)]* III; *puf-11(q971) puf-3(q966)/puf-11(q971) tmC9[F36H1.2(tmIs1221)]* IV
DG5673	*+/hT2[umnIs60(myo-2p::mKate2)]* I; *let-711(tn2136)/hT2[bli-4(e937)]* III; *puf-11(q971) puf-3(q966)/puf-11(q971) tmC9[F36H1.2(tmIs1221)]* IV
DG5674	*+/hT2[umnIs60(myo-2p::mKate2)]* I; *let-711(tn2137)/hT2[bli-4(e937)]* III; *puf-11(q971) puf-3(q966)/puf-11(q971) tmC9[F36H1.2(tmIs1221)]* IV
DG5675	*+/hT2[umnIs60(myo-2p::mKate2)]* I; *let-711(tn2138)/hT2[bli-4(e937)]* III; *puf-11(q971) puf-3(q966)/puf-11(q971) tmC9[F36H1.2(tmIs1221)]* IV
DG5680	*wrdSi18[mex-5p::TIR1::F2A::mTagBFP2::tbb-2 3'UTR]* I; *ccf-1(tn2132[ccf-1::gfp::aid::3xflag])* III
DG5682	*wrdSi18[mex-5p::TIR1::F2A::mTagBFP2::tbb-2 3'UTR]* I; *let-711(tn2130[let-711::gfp::aid::3xflag])* III
DG5701	*+/hT2[umnIs60(myo-2p::mKate2)]* I; *let-711(tn2135)/hT2[bli-4(e937)]* III
DG5702	*+/hT2[umnIs60(myo-2p::mKate2)]* I; *let-711(tn2136)/hT2[bli-4(e937)]* III
DG5703	*+/hT2[umnIs60(myo-2p::mKate2)]* I; *let-711(tn2137)/hT2[bli-4(e937)]* III
DG5704	*+/hT2[umnIs60(myo-2p::mKate2)]* I; *let-711(tn2138)/hT2[bli-4(e937)]* III
DG5713	*ccf-1(gk40)/tmC29[unc-49(tmIs1259)]* III
DG5728	*ccf-1(tn2166)/tmC29[unc-49(tmIs1259)]* III
DG5730	*ccf-1(tn2167)/tmC29[unc-49(tmIs1259)]* III
DG5732	*ccf-1(tn2168)* III
DG5751	*+/hT2[umnIs60(myo-2p::mKate2)]* I; *ccf-1(tn2166)/hT2[bli-4(e937)]* III; *puf-11(q971) puf-3(q966)/puf-11(q971) tmC9[F36H1.2(tmIs1221)]* IV
DG5752	*+/hT2[umnIs60(myo-2p::mKate2)]* I; *ccf-1(tn2167)/hT2[bli-4(e937)]* III; *puf-11(q971) puf-3(q966)/puf-11(q971) tmC9[F36H1.2(tmIs1221)]* IV
DG5754	*ccf-1(tn2168)* III; *puf-11(q971) puf-3(q966)/puf-11(q971) tmC9[F36H1.2(tmIs1221)]* IV
DG5755	*+/hT2[umnIs60(myo-2p::mKate2)]* I; *ccf-1(gk40)/hT2[bli-4(e937)]* III; *puf-11(q971) puf-3(q966)/puf-11(q971) tmC9[F36H1.2(tmIs1221)]* IV
DG5779	*lin-41(tn1541[gfp::s-tag::lin-41] tn2200 [smFoxΔ in 3'UTR]* I
DG5919	*lin-41(tn2238[smFoxΔ in 3'UTR])* I
DG5965	*lin-41(tn2238[smFoxΔ in 3'UTR])* I; *spn-4(tm291)/tmC3[egl-9(tmIs1230) spn-4(tn2056[spn-4::gfp::3xflag])]* V
DG5972	*chs-1(tn2255[FoxΔ in 3'UTR])* I
DG5974	*ccr-4(tm1312)/tmC9[F36H1.2(tmIs1221)]* IV
DG6008	*ccf-1(tn2168)* III; *ccr-4(tm1312)/tmC9[F36H1.2(tmIs1221)]* IV
DG6272	*lin-41(tn1541[gfp::s-tag::lin-41] tn2200 [smFoxΔ in 3'UTR]* I; *sel-10(ok1632)* V

[a] EMS suppressor strain used for WGS; 1x backcross to DG5365.
[b] EMS suppressor strain used for WGS; 3x backcross to DG5365.
[c] EMS suppressor strain used for WGS; 2x backcross to DG5365.
[d] EMS suppressor strain used for WGS; 5x backcross to DG5365.

Table S3. Sequence of oligonucleotides used in this study for genome editing, PCR, sequencing and plasmid constructions.

Available for download at
https://journals.biologists.com/dev/article-lookup/doi/10.1242/dev.205295#supplementary-data

Table S4. Gene Ontology (GO) output. (Related to Fig. S4).

Available for download at
https://journals.biologists.com/dev/article-lookup/doi/10.1242/dev.205295#supplementary-data

Table S5. scRNA-seq categories. (Related to Figs 2, S5 and S6).

Available for download at
https://journals.biologists.com/dev/article-lookup/doi/10.1242/dev.205295#supplementary-data

Table S6. Probesets used for smFISH and smiFISH. (Related to Figs 3-8,12, S7-12, S17 and S18).
ipt counts and statistics for *lin-41* and *set-3* mRNA in individual blastomeres. (Related to Fig. 5).

Available for download at
https://journals.biologists.com/dev/article-lookup/doi/10.1242/dev.205295#supplementary-data

Table S7. Transcript counts and statistics for *lin-41* and *set-3* mRNA in individual blastomeres. (Related to Fig. 5).

Available for download at
https://journals.biologists.com/dev/article-lookup/doi/10.1242/dev.205295#supplementary-data

Table S8. Transcript counts for comparing the wild type and *spn-4(tm291)* mutants—raw values and statistics. (Related to Figs 3 and S8).

Available for download at
https://journals.biologists.com/dev/article-lookup/doi/10.1242/dev.205295#supplementary-data

Table S9. Transcript counts, statistics and n values for gonad smFISH experiments (Related to Figs 4 and S9).

Available for download at
https://journals.biologists.com/dev/article-lookup/doi/10.1242/dev.205295#supplementary-data

Table S10. Transcript counts for analyzing *lin-41* 3'UTR deletions—raw values and statistics. **(**Related to Figs 6 and S10).

Available for download at
https://journals.biologists.com/dev/article-lookup/doi/10.1242/dev.205295#supplementary-data

Table S11. Transcript counts for analyzing the genetic interaction between a *lin-41* Rbfox-motif deletion and *spn-4* mutants —raw values and statistics. (Related to Figs 7 and S11).

Available for download at
https://journals.biologists.com/dev/article-lookup/doi/10.1242/dev.205295#supplementary-data

Table S12. Transcript counts for analyzing the Rbfox-motif deletion in the *chs-1* 3'UTR deletion—raw values and statistics. (Related to Fig. 8).

Available for download at
https://journals.biologists.com/dev/article-lookup/doi/10.1242/dev.205295#supplementary-data

Table S13. Quantitative analysis of GFP::LIN-41 expression in wild-type and mutant embryos. (Related to Fig. 9 and S12B).

Available for download at
https://journals.biologists.com/dev/article-lookup/doi/10.1242/dev.205295#supplementary-data

Table S14. Transcript counts for analyzing the requirement of LET-711 and CCF-1 for SPN-4-dependent maternal mRNA clearance—raw values and statistics for controls and experimentals. (Related to Figs 12, S7B and S18).

Available for download at
https://journals.biologists.com/dev/article-lookup/doi/10.1242/dev.205295#supplementary-data

Table S15. Transcript counts and statistics for comparing *lin-41* transcript levels in wild-type and *sel-10(ok1632)* null mutant embryos. (Related to Fig. S12).

Available for download at
https://journals.biologists.com/dev/article-lookup/doi/10.1242/dev.205295#supplementary-data

Development • Supplementary information
