# Supplementary material for: The SPN-4 Rbfox RNA-binding protein selects maternal mRNAs for CCR4-NOT-dependent clearance in early Caenorhabditis elegans embryos
Source: Development. 2026 Jun 4;153(11):dev205295. doi: 10.1242/dev.205295 (PMC13286378; doi:10.1242/dev.205295)
Supplement: Supplementary file 1 [file develop-153-205295_review_history.pdf]

The SPN-4 Rbfox RNA-binding protein selects maternal mRNAs for CCR4-NOT-dependent clearance in early *Caenorhabditis elegans* embryos

Caroline A. Spike, Dylan M. Parker, Tatsuya Tsukamoto, Naly Torres Mangual, Erika C. Tsukamoto, Karissa Coleman, Micah D. Gearhart, David Greenstein and Erin Osborne Nishimura

DOI: 10.1242/dev.205295

Editor: Swathi Arur
Review timeline
Original submission: 8 October 2025
Editorial decision: 10 November 2025
First revision received: 1 April 2026
Editorial decision: 20 April 2026
Second revision received: 29 April 2026
Accepted: 30 April 2026

Original submission

First decision letter

MS ID#: dev.205295

MS TITLE: The SPN-4 Rbfox RNA-binding protein selects maternal mRNAs for CCR4-NOT-dependent clearance in early Caenorhabditis *elegans embryos*

AUTHORS: Caroline A. Spike, Dylan M. Parker, Tatsuya Tsukamoto, Naly Torres-Mangual, Erika C. Tsukamoto, Karissa Coleman, Micah D. Gearhart, David Greenstein and Erin Osborne Nishimura

Dear Dr Osborne Nishimura,

I have now received all the referees' reports on the above manuscript, and have reached a decision. The referees' comments are appended below.

As you will see, the referees express considerable interest in your work and generally agree with the strengths in the study. However, as you will note, reviewer 3 is less enthusiastic, and identifies additional experiments to fortify the rigor in the study, including inclusion of some controls. Overall, the three reviewers recommend some textual changes, and as suggested by reviewer 3 additional experimental analysis. If you are able to revise the manuscript along the lines suggested, which involve further experiments, I will be happy receive a revised version of the manuscript. Your revised paper will be re-reviewed by one or more of the original referees. Please also note that Development will normally permit only one round of major revision.

Reviewer 1

Advance summary and potential significance to field

This manuscript describes the characterization of the Rbfox1 RNA-binding family member SPN-4 and how it regulates maternal mRNAs through a CCR4-NOT- dependent mechanism. The authors first

© 2026. Published by The Company of Biologists under the terms of the Creative Commons Attribution License (https://creativecommons.org/licenses/by/4.0/).

start by performing IPs to evaluate which mRNAs SPN-4, LIN-41 and OMA1 interact with. Overlap was greatest between the mRNAs associated with LIN-41 and OMA-1, while SPN-4 only shared 4-14% overlap with the other two proteins. Further analysis of the 728 SPN-4-associated mRNAs revealed that many decreased in abundance in somatic cells over time. smFISH experiments showed that loss of spn-4 resulted in increased levels of SPN-4- associated transcripts, indicating that SPN-4 likely plays a role in regulating the stability of these RNAs. SPN-4 protein binds to Rbfox motifs in the 3'UTRs of target transcripts and deletion of these motifs in specific mRNAs results in their elevation in vivo. The authors next performed a genetic screen and found that several components of the CCR4 complex act as effectors of SPN-4 in maternal mRNA clearance. Lastly, the authors provide evidence that loss of the CCR4-NOT complex stabilizes RNAs targeted by SPN-4.

Overall, this is an extremely well-written and elegant study that provides compelling evidence that SPN-4 regulates the clearance of maternal RNA. The data are robust, beautifully presented, and generally support the authors' conclusions. The paper is certainly appropriate for Development. However, several points should be addressed prior to publication:

Comments for the author

1) Data presented in Figure 3C for chs-1 and cpg-2 smFISH are duplicate images (both WT and spn-4 mutants). I imagine this represents a simple and innocent oversite. Please provide the correct images and double check that there are no other duplicated images throughout the figures (I did not find any).

2) Quantification of the mRNA abundance in various mutant backgrounds is based on fluorescent intensity of smFISH staining. I appreciate that many of these RNAs may be low abundance, but orthogonal methods for quantifying mRNA levels, such as qRT-PCR, RNA-seq and/or northern blot would greatly bolster the strength of the conclusions. This does not have to be done for every transcript analyzed, but a couple of example mRNAs would go a long way.

3) Along the same lines, the authors often express mRNA levels as number of transcripts. Were spike-ins or other controls run to ensure that fluorescence always tightly correlated with transcript number?

4) The initial RNA-IP experiments were not done in the presence of cross-linker (formaldehyde or UV) based on the description of the technique in the Material and Methods. RNA-binding proteins will often show spurious interactions with RNAs once cells are broken open. The only way to ensure that proteins truly interact with RNAs in vivo is to perform crosslinking before cell lysis. Addressing this point is important before making claims regarding specific interactions. Given the follow-up data, I trust that SPN-4 targets specific RNAs for degradation, but this point still stands.

5) In Figure 4B, the lin-41 mRNA signal for the Δ777-1054 deletion looks lower throughout the embryo in the 4-cell image provided. In addition, the signal appears in large puncta. Do the authors have an explanation for this pattern? Is this typical for this specific construct?

6) This may be beyond the current scope of the paper, but pulse-labeling experiments designed to measure mRNA half-lives +/- spn-4 would enhance the conclusions of the story.

<u>Reviewer 2</u>

Advance summary and potential significance to field

In figure 1, the authors use IP followed by mRNA sequencing to identify transcripts associated with SPN4, LIN41 and OMA1 in oocytes that cannot be fertilized, so they cannot degrade maternal mRNAs. They find mRNA associated with SPN4 with eggshell genes, cell cycle and sexual reproduction/gamete formation. In figure 2, SPN4 transcripts are degraded in the embryo from 2-16 cells stage, while transcripts associated with OMA1 or LIN41 are not. This suggests that SPN4 is specifically required to downregulated maternal mRNAs. The exception is the P-lineage which is transcriptionally silent, but also D, which they do not comment on. The two clusters from tintori et al that correspond to degraded mRNAs overlap with SPN4 associated mRNAs. Cluster 5 was

© 2026. Published by The Company of Biologists under the terms of the Creative Commons Attribution License (https://creativecommons.org/licenses/by/4.0/).

statistically significant for SPN4 association while Cluster 3 was significantly associated with SPN4, LIN41 and OMA1. SPN4 is also specifically under enriched for association with zygotic mRNAs. Overall, SPN4 association accounts for ~11% of maternally degraded mRNAs. To confirm the genomic data, in Figure 3 the authors perform smFISH on 12 SPN4 associated targets, 4 OMA1/LIN41 targets and 4 control targets in control versus spn4 mutants. 9 of the 12 SPN4 targets are significantly upregulated while only 1 of the controls is specifically upregulated. This provides further evidence that SPN4 is required to degrade maternal mRNAs. In figure 4, the authors show that mutations in the 3' UTR that affect an Rbfox binding site (which is the top enriched motif-figure 6E) preclude the normal degradation of lin-41 RNA, raising the possibility that Rbfox binding is required for the degradation of maternal mRNAs. In figure 5, the authors show that the increase in lin41 transcript abundance seen in spn4 mutants is not synergistic with the increase seen in the RBfox binding site mutations. This is consistent with SPN4 functioning through this binding site. In figure 6, the authors find the same thing for chs-1 RNA. In figure 7, the authors demonstrate the binding of SPN4 directly to the Rbfox motif in lin41 and to the chs-1 RNA.
PUF3 and PUF11 mediate translational suppression of SPN4. In a double mutant SPN4 is prematurely expressed in oocytes. Thus, it is possible that the sterility of PUF double mutants is due to premature clearance of maternal transcripts. Consistent with this possibility, the authors find that partial loss of SPN4 slightly suppresses the embryonic lethality caused by the PUF double mutant and in the PUF double there is an increase in chs-1 transcripts and accelerated meiosis. The authors also isolated spn4 mutations in an unbiased PUF double mutant EMS screen for suppressors of sterility. Taken together, these results suggest that the sterility of PUF double mutants is due to the premature clearance of maternal transcripts. To identify putative effectors of SPN4 maternal mRNA clearance, the authors also identified other suppressors of the PUF double mutant sterility. let711 and ccf1 loss of function alleles were both identified in this suppressor screen and confirmed by CRISPR in the PUF double mutant. In addition, both LET711 and CCF1 were shown to be required for the degradation of lin41 and chs-1 RNA, as well as mRNA in general. Together these data implicate the CCR4-NOT deadenylase complex in maternal mRNA degradation. Finally, the authors show that a second deadenylase complex containing CCR4 is also required for degradation of maternal lin41 and chs-1 RNA and seems to function redundantly with CCF-1.
Overall, this is a truly outstanding paper with tons of high quality data that is well described and beautifully presented. As described in the summary above, the data have major mechanistic implications for our understanding of how maternal mRNAs are degraded. To be completely honest, in my opinion the data presented here could be two Development papers. I don't believe there is a need for any new experiments, only a few minor clarifications to points below.

Comments for the author

In figure 3E, the meaning of the dashed line and NA is unclear. Also, the 1 control that is listed as significant in the text is not labelled in the figure. If it is Y37H2A, why is that significant? If it is just increased and not significant, why not? And why is R05H11.1 significant, when other targets like vab-2 and mex-5 are not? I assume this has to do with the individual N. To clarify this, it might be important to list the actual N for each target rather than describing an overall N in general, as the authors have done in the subsequent figures.
In figure 4B, when there is less degradation of the lin41 RNA, particularly in the Med and Lg foxΔ, it seems like there is an accumulation of RNA in P-granules, which is potentially interesting. This is also true for chs-1 RNA in figure 6B. Could this potential concentration of RNA into P-granules be concentration dependent? The authors should potentially comment on this or try a P-granule marker to confirm this, but this is not necessary for this publication.
In figure 4, even though there is a clear elevation of lin41 RNA in the fox deletions compared to WT, there is still a substantial decrease from the 2-4 cell stage. Is this due to normal turnover? Or due to another mechanism of degradation? The authors should comment on this.
In figure 5B, I assume that the right side is the 4-cell stage (it is labelled as the 2-cell stage, identical to the left side, so the labelling should be fixed). If so, there is now no increase in lin41 RNA in the sm fox deletion at the 4-cell stage like there was in figure 4C. Also, if there is no increase in this experiment, the image in 5A does not appear to be representative since it looks like there is an increase. The authors should probably comment on this.
In figure 7, the authors don't test an Rbfox binding site mutation in chs-1. While this is probably ok for the overall model, it does leave the slight impression that they may have tried the mutation and it did not show the expected result. I am assuming this is not the case, but the authors might want

© 2026. Published by The Company of Biologists under the terms of the Creative Commons Attribution License (https://creativecommons.org/licenses/by/4.0/).

to consider performing this experiment, or somehow explicitly stating this, so that readers don't worry about that possibility.
I don't think figure 6F is referenced in the text.
The quantification in figure 8H is not great. The chs-1 transcripts levels in WT are compared to WT. Does that mean that they are all identical? Presumably, you just need to quantify the mutant compared to the WT average, but this should be clarified. 75% of 18 is 13.5, so presumably the 75% is out of the 12 embryos plus oocyte count. But since it doesn't match the number of gonads sampled, does that mean some of N=18 were not sampled? In that case why are they included in the N? In any case, it is a bit confusing, so needs to be clarified. It also might be helpful to more clearly describe the PUF double mutant phenotype. Additionally, the supplemental figure legend states that mating was performed to match the meiotic maturation rate of WT and it shows rescued maturation but the reason for the mating is a bit vague (perhaps owing to the lack of description of the PUF double mutant phenotype. At a minimum the reason for the mating should be described in the text, not just the figure legend. Overall, I believe the result, but the data are difficult to follow and the quantification is not ideal.
It would be helpful if the authors provided more introduction of the CCR4-NOT deadenylase complex, including LET711, CCF1 and CCR4
I believe that figure 9F is not cited in the text.
In figure 10, depletion of LET711 appears to block mRNA degradation, while depletion of CCF1 seems to more delay degradation. The authors should comment on this. Also, there is no corresponding image of CCR-4. Ideally this would be shown.
I hate to even suggest this, because of all the work that has already been done, but a simple model figure would be helpful.
This question is not relevant to this current paper or the review, but I think it would be interesting to ask, does this system only affect maternal RNAs? Or does it also affect any siRNAs?

Reviewer 3

Advance summary and potential significance to field

This paper reports on a potential role for the Rbfox-related RNA binding protein SPN-4 in RNA degradation in C. elegans embryos. Several studies have reported that SPN-4 binds to specific sequences in 3' UTRs to regulate the translation of maternal mRNAs in C. elegans embryos. In this paper, the authors identify hundreds of SPN-4-bound mRNAs in oocytes and show that 12 appear to decay more slowly in spn-4 mutant embryos as detected by situ hybridization. For two transcripts, the authors show that deletion of the predicted SPN-4 binding sites also slows down decay. The authors also show that knock-down of the CCR4-Not deadenylase also slows down the decay of two SPN-4 bound transcripts. Based on these observations, the authors conclude that "SPN-4 selects maternal mRNAs for CCR-4/NOT dependent clearance".

This conclusion is premature. SPN-4 is highly expressed in oocytes and P blastomeres where maternal mRNAs are stable, so it's unlikely that SPN-4 binding is sufficient to trigger decay. Furthermore, prior studies have shown that SPN-4 regulates the translation of maternal mRNAs. A likely possibility is that SPN-4 binding, together with other RNA-binding proteins, tunes the translational rate of maternal mRNAs, which in turn can impact decay rate depending on the specific transcript, cell type and developmental stage. For example, certain mRNAs could appear stabilized in spn-4 mutants simply because their translation rate is increased compared to wild-type. The data presented are not sufficient to rule out this model and are not sufficient to support the claim that "SPN-4 selects mRNAs for maternal clearance". In addition, there are several technical concerns (see specific comments). Most importantly, the experiments shown in Fig. 1 (IP experiments that define SPN-4-bound transcripts) are foundational yet lack a critical control, and thus undermine the premise for the entire paper.

https://doi.org/10.1016/j.ydbio.2012.01.002, https://doi.org/10.1242/dev.00469, https://doi.org/10.1016/j.gene.2014.10.042

Comments for the author

Specific comments:

© 2026. Published by The Company of Biologists under the terms of the Creative Commons Attribution License (https://creativecommons.org/licenses/by/4.0/).

Fig. 1: The IP experiment lacks a control using the FLAG beads on a lysate where SPN-4 is not tagged. As shown now, enrichment over input does not control for antibody/bead specificity. The authors should also show expression of SPN-4 in the feminized worms used for the IP.

Fig. 2: The plot shown should be replaced with that in Fig. S3E and the behavior of each lineage should be shown separately. The number of genes in each category should be indicated in the figure. This figure should also show a graph for all maternal mRNAs degraded in that time frame for comparison, to make it clear that SPN-4-bound mRNAs are a fraction of that cohort. Each plot should show the data for each gene (scatter plot style) - rather than the average for that gene group - to show the variance within each group. Are there SPN-4 bound mRNAs that are not degraded? Is the decay behavior of SPN-4-bound transcripts similar to that observed for most maternal mRNAs?
The no-association mRNA category exhibit very low starting levels? This seems odd. The SPN-4 associated mRNAs in contrast are among the most abundant? Does that suggest that association with SPN-4 stabilizes maternal mRNAs in oocytes or zygotes?

Fig. 3A: Why was the spn-4 GFP introduced on a balancer chromosome?? Balancer chromosomes are usually used to suppress recombination but that does not seem the purpose there. Does the balancer chromosome also have a lethal mutation to prevent the propagation of spn-4::GFP homozygotes??

Fig. 3D: Most transcripts exhibit higher levels in spn-4 at the very first stage tested (2-cell). What is the evidence that SPN-4 acts in embryos, as opposed to oocytes or even an earlier stage during germline development?

Fig. 8: The inference that CCR4-NOT are candidate effectors of SPN-4 mediated degradation is not supported by data showing direct interactions between the two or a specific effect of CCR4-NOT on spn-4-bound transcripts. CCR4-NOT has already been reported to be the main deadenylase in the C. elegans germline, so it is likely to affect all maternal mRNAs??
https://pubmed.ncbi.nlm.nih.gov/23843623/

All figures: The authors should adopt a consistent color scheme throughout and report the data using superplots where individual data points are shown (instead of averages) and color coded to show replicates where applicable.

First revision

Author response to reviewers' comments

We thank the reviewers and the editor for the constructive suggestions. We have addressed all points raised. We have included a pdf file of the manuscript in which changes in response to the reviewers' comments are highlighted. For ease of presentation, we have divided our response into two sections: (I) Critiques Requiring New Experiments or Data Analysis; and (II) Critiques Requiring Textual Changes, Clarification, or Presentational Elements.

I. Critiques Requiring New Experiments or Data Analysis

1. Reviewer #1. The initial RNA-IP experiments were not done in the presence of cross-linker (formaldehyde or UV) based on the description of the technique in the Material and Methods. RNA-binding proteins will often show spurious interactions with RNAs once cells are broken open. The only way to ensure that proteins truly interact with RNAs in vivo is to perform crosslinking before cell lysis. Addressing this point is important before making claims regarding specific interactions. Given the follow-up data, I trust that SPN-4 targets specific RNAs for degradation, but this point still stands.

© 2026. Published by The Company of Biologists under the terms of the Creative Commons Attribution License (https://creativecommons.org/licenses/by/4.0/).

The Reviewer is correct. Our methods do not utilize crosslinking. We use non-denaturing conditions because our chief interest is in elucidating what different classes of oocyte RNPs do at a biological level *in vivo*. This requires a knowledge of the full set of proteins and mRNAs associated with a given RNA-binding protein.

For this reason, we do not claim that any isolated RNA-binding protein (e.g., SPN-4, LIN-41, OMA-1) directly binds a specific transcript, but rather that the transcript is "associated" with that particular RNA-binding protein. Downstream analyses, such as those found in this manuscript for the *Lin-41* and *chs-1* mRNAs, are needed to establish the functional relevance of the association and whether the association is likely to be direct. We have emphasized this distinction by adding a sentence to the Materials and Methods section,

"Because our RNP isolation procedures do not utilize cross-linking or denaturing conditions, we refer to the isolated transcripts as "associated" with a given RNA-binding protein (e.g., "SPN-4-associated"), with downstream analyses required to define the functional consequences and determine whether the association is direct."

We note that our approach has proven useful and informative for the field, as highlighted by the biologically relevant information we have gleaned from oocyte RNP purifications and the many publications that utilize our reagents or cite our results (some examples are: Theil et al., 2019; Lee et al., 2020; Albarqi and Ryder, 2021; Gajjar et al., 2025; Shukla et al., 2025). In prior work, we found that immunopurified oocyte RNPs are stable on glycerol gradients (Tsukamoto et al., 2017) and contain several RNA-binding proteins in addition to the one used for isolation. Perhaps because the RNPs are large, stable entities, isolated from a single tissue in which they are functional, non-specific trapping of extraneous transcripts seems to be minimal. For example, for SPN-4 immunopurifications, among the 100 most abundant transcripts in the extract, only 5 biologically meaningful ones (*cbd-1*, *cpg-1*, *cpg-2*, *vig-1*, *ndk-1*) were significantly enriched in the purifications. Possibly, the RNA-technical ine point is that our method of elution by TEV cleavage, provides an added specificity step. We do observe what appears to be some minimal non-specific adsorption of mRNAs to TEV-eluted RNPs as shown in Fig. S3. We control for this by measuring the fold enrichment compared to the lysate and doing appropriate statistical tests. When prioritizing a possible target for detailed analysis, we also consider its relative abundance in the RNP purification and likely biological relevance.

2. Reviewer #2. In figure 3E, the meaning of the dashed line and NA is unclear. Also, the 1 control that is listed as significant in the text is not labelled in the figure. If it is Y37H2A, why is that significant? If it is just increased and not significant, why not? And why is R05H11.1 significant, when other targets like vab-2 and mex-5 are not? I assume this has to do with the individual N. To clarify this, it might be important to list the actual N for each target rather than describing an overall N in general, as the authors have done in the subsequent figures.

Thank you for the comment. We have presented the data in a new way, now in Fig. 3E and 3F using statistical methods that encompass all the genes shown. We tested 19 mRNAs for mRNA abundance by smiFISH with and without maternal *spn-4* activity. To correlate the smFISH and immunoprecipitation indings, the significant increase in mRNA transcripts in *spn-4(tm291)* null mutants versus SPN-4 (+) (N2) strains, calculated as -log10 p-value for a one-tailed *t*-test (BH adjusted), was plotted against the RNA-seq IP enrichment values (from Fig. 1 and Table S1) for SPN-4 associated and independent mRNAs in Fig. 3E. Fig. 3F shows heatmaps comparing smFISH significance, IP-enrichment for various RNA-binding proteins and expression levels.

3. Reviewer #2. In figure 7, the authors don't test an Rbfox binding site mutation in chs-1. While this is probably ok for the overall model, it does leave the slight impression that they may have tried the mutation and it did not show the expected result. I am assuming this is not the case, but the authors might want to consider performing this experiment, or somehow explicitly stating this, so that readers don't worry about that possibility.

© 2026. Published by The Company of Biologists under the terms of the Creative Commons Attribution License (https://creativecommons.org/licenses/by/4.0/).

Thank you for bringing this issue to our attention. We have done the requested binding experiment with a *chs-1* probe containing base substitutions in the Rbfox motif. The results are shown in Fig. S16B and are summarized in Fig. S16D and Fig. 10C (line plot #8). The results shows that the SPN-4 RRM only exhibits non-specific and non-saturable binding to the control probe as expected.

4. Reviewer #2. The quantification in figure 8H is not great. The chs-1 transcripts levels in WT are compared to WT. Does that mean that they are all identical? Presumably, you just need to quantify the mutant compared to the WT average, but this should be clarified. 75% of 18 is 13.5, so presumably the 75% is out of the 12 embryos plus oocyte count. But since it doesn't match the N? In any case, it is a bit confusing, so needs to be clarified. It also might be helpful to more clearly describe the PUF double mutant phenotype. Additionally, the supplemental figure legend states that mating was performed to match the meiotic maturation rate of WT and it shows rescued description of the PUF double mutant phenotype. At a minimum the reason for the mating should be described in the text, not just the figure legend. Overall, I believe the result, but the data are dificult to follow and the quantification is not ideal.

Thank you for the comment. We have fixed these issues by conducting additional experiments. We have now conducted smFISH in dissected gonads in which we segmented oocytes using WGA staining to get accurate counts of *chs-1* and *Lin-41* transcripts in oocytes. This new quantitative data used unmated hermaphrodites. These new results are shown in Fig. 4 and Fig. S9.

5. Reviewer #3: "SPN-4 is highly expressed in oocytes and P blastomeres where maternal mRNAs are stable, so it's unlikely that SPN-4 binding is suficient to trigger decay."

Thank you for the comment. We are not claiming that SPN-4 association is sufficient to trigger decay. Rather, cellular context is crucial: what we observe is that SPN-4-mediated mRNA clearance occurs in the somatic blastomeres of the early embryo. For example, we purified SPN- 4-associated mRNAs from the oogenic germline where these mRNAs are apparently stable.
Likewise, SPN-4 accumulates at high levels in the germline blastomeres where some notable SPN-4-associated mRNAs (e.g., *nos-1* and *nos-2*) are stable. We appreciate that we failed to make this distinction in our initial submission and thank the reviewer for pointing out this deficiency.

We have rectified this omission by providing two new lines of experimental data in a new section called, "SPN-4-dependent mRNA clearance occurs in the somatic blastomeres of the early embryo." In the first line of evidence, we examine *Lin-41* and/or *chs-1* transcript levels in dissected gonads of the wild type, *spn-4(tm291)* null mutants, and *puf-3111Δ* mutants. The data, now in Fig. 4 and Fig. S9, support the conclusion that SPN-4-dependent mRNA clearance occurs after fertilization. Importantly, transcript levels are no different in SPN-4-expressing and -non- expressing oocytes.

In the second line of evidence, we investigated whether SPN-4-dependent mRNA clearance is spatially regulated in the embryo. We reanalyzed our *Lin-41* smFISH data at the 2- and 4-cell stages with cell-specific resolution to assess *Lin-41* transcript concentration in AB, P_1, ABa, ABp, EMS, and P_2 cells. The data, now in Fig. 5, show that *Lin-41* transcripts were significantly less concentrated in the somatic blastomere AB than in its germline sister P_1, whereas the *set-3* mRNA control showed no marked AB-P_1 asymmetry. After AB divides, *Lin-41* transcripts declined sharply in ABa and ABp synchronously, indicating robust clearance in the AB lineage. By contrast, the P_1 daughters displayed asymmetric Lin-41 levels, with the somatic blastomere EMS containing substantially fewer transcripts than the germline blastomere P_2. These data argue that SPN-4-dependent clearance is preferentially activated in somatic blastomeres and attenuated or prevented in germline blastomeres.

Our manuscript focuses on reporting the role of SPN-4 in promoting maternal mRNA clearance shortly after fertilization, providing a key part of the biological mechanism that explains the phenomenon first observed by Stoeckius et al. (2014). How this mechanism might be "turned

© 2026. Published by The Company of Biologists under the terms of the Creative Commons Attribution License (https://creativecommons.org/licenses/by/4.0/).

off" or attenuated in germline blastomeres is beyond the scope of this initial report. Yet, as we mention in the Discussion, there is precedent for this mode of regulation by DrosophiLa Smaug, which participates both in maternal mRNA clearance and translational repression.

6. Reviewer #3. "Certain mRNAs could appear stabilized in *spn-4* mutants simply because their translation rate is increased compared to the wild-type."

Thanks for raising this important issue. To test whether the targets of SPN-4 have increased rates of translation, we examined GFP::LIN-41 protein levels in *spn-4* null or small *FoxΔ* genetic backgrounds. Because LIN-41 is inactivated and degraded in a seL-10-dependent manner upon the onset of oocyte meiotic maturation (Spike et al., 2018, 2022), these experiments were conducted in the *seL-10(ok1632)* null genetic background where any accumulation of GFP::LIN- 41 could be assessed. *seL-10* null mutants are viable and fertile, and SPN-4-dependent maternal mRNA clearance is not impacted by the mutation (we show these data in Fig. S12). Our results are consistent with the view that GFP::LIN-41 is not translated at an increased rate in early embryos of spn-4 null mutants or upon deletion of the Rbfox motif. The data are presented in Figure 9 and the new section reads,

"The observation that SPN-4 functions through the Rbfox motif to reduce *Lin-41* transcript levels ~10-fold in somatic blastomeres-together with evidence that it impacts early embryonic gene expression (Gomes et al., 2001; Huang and Hunter, 2015; Huang et al., 2002; Ogura et al., 2003) and can act as a translational repressor (Jadhav et al., 2008; Oldenbroek et al., 2012, 2013)- prompted us to assess *spn-4* null mutant and *FoxΔ*) backgrounds for effects on GFP::LIN-41 protein levels in early embryos. Upon meiotic maturation onset, LIN-41 undergoes SCF^{SEL-10} E3 ubiquitin ligase-mediated degradation (Spike et al., 2018; Spike et al., 2022). We thus quantified GFP::LIN-41 in the viable, fertile seL-10(ok1632) null background, where ubiquitin-mediated degradation of LIN-41 is blocked but maternal Lin-41 mRNA clearance remains unaffected (Fig. S12). Unlike Lin-41 mRNA levels, GFP::*LIN-41* protein was not markedly elevated in *spn-4* null or small *FoxΔ*) backgrounds (Fig. 9). This suggests *SPN-4*-mediated *Lin-41* transcript clearance occurs in the early embryo when these transcripts are not robustly translated."

We would like to point out that the mechanism we are reporting makes sense in the biological context. The clearance of *lin-41* mRNA by SPN-4, after LIN-41 has completed its oogenic functions, represents a reinforcing mechanism for the regulation of LIN-41 because the SCF^{SEL-10} removes LIN-41 protein in response to CDK-1 activation during meiotic maturation. In a similar vein, many proteins represented by SPN-4-associated mRNAs participate in eggshell formation (e.g., *chs-1*, *cbd-1*, *cpg-1*, *cpg-2*, etc.) and are eliminated shortly thereafter. For these genes, it makes biological sense why the corresponding proteins and transcripts would be eliminated after eggshell formation.

We would also like to emphasize that our new model for SPN-4 function in maternal mRNA clearance is compatible with prior results in the literature. SPN-4 was first described by the Bowerman lab as a maternal RRM-containing protein required for the proper pattern of embryonic cleavages (Gomes et al., 2001). They suggested that "SPN-4 post-transcriptionally with the pleiotropic and early manifesting defects observed in *spn-4* null mutants. Our study identifies SPN-4 targets genome-wide and demonstrates post-transcriptional regulation of many of the genes we studied. Ogura et al., (2003) found SPN-4 as a two-hybrid interactor of the CCCH zinc-inger protein POS-1 (incidentally, POS-1 is recovered in SPN-4 tandem affinity purifications; our unpublished results). They provided evidence that GLP-1 protein is ectopically expressed in posterior blastomeres (EMS and P2) in *pos-1* mutants. Further they found that GLP-1 protein was not produced in early embryos of *spn-4* null mutants. Their study did not present data that shows SPN-4 functions as a translational repressor. They did show, using a three-hybrid interaction, that SPN-4 can interact with sequences in the *gLp-1* 3'UTR. We note that these sequences do not contain Rbfox motifs, and we did not identify *gLp-1* as a SPN-4-associated transcript (Table S1).
How SPN-4 promotes *gLp-1* expression in the embryo is unclear. Ogura et al. (2003) did not examine the effects of SPN-4 on *gLp-1* mRNA levels and neither did we. Jadhav et al.,

© 2026. Published by The Company of Biologists under the terms of the Creative Commons Attribution License (https://creativecommons.org/licenses/by/4.0/).

(2008) showed that SPN-4 suppresses the translation of *nos-2* mRNA in germline blastomeres such that it is first expressed at the 28-cell stage in P4 (Jadhav et al., 2008). This study also demonstrated binding of GST-SPN-4 to the *nos-2* 3'UTR, though binding affinities were not Nishimura group (Parker et al., 2020) showed that both the *spn- 4(or191ts)* mutation and *spn-4(RNAi)* result in increased levels of *nos-2* transcripts in early embryos. In the present study, we found *nos-2* as an mRNA associated with SPN-4, OMA-1, and LIN-41, which is also consistent with the observation that OMA-1/2 represses translation of *nos- 2* in oocytes (Jadhav et al., 2008). Oldenbroek et al., (2012) provided evidence that SPN-4 could repress translation of *zif-1* in EMS and P2. They did not examine the effects of SPN-4 on *zif-1* transcripts and neither did we. In our study, we identified *zif-1* as a transcript associated with SPN-4, OMA-1, and LIN-41. This its well with the observations that OMA-1/2 and LIN-41 repress translation of ZIF-1 in oocytes (Guven-Ozkan et al., 2010; Spike et al., 2014). Craig Hunter's lab found that SPN-4 afects the accumulation of the KH RNA-binding protein MEX-3 such that MEX- 3 protein levels were higher following *spn-4(RNAi)* than in the wild-type and persisted to the 14- to 18-cell stage (Huang et al., 2002; Huang and Hunter, 2015). These authors favored a model in which SPN-4 promotes MEX-3 protein degradation; however, these studies didn't examine *mex-3* mRNA levels. In our study, we found *mex-3* as a transcript associated with SPN-4 and LIN-41, and we note that Stoeckius et al. (2014) described *mex-3* as a transcript subject to early decay.
Thus, these data would be consistent with a model in which SPN-4 might promote the clearance of *mex-3* mRNA. We previously reported that SPN-4 could repress *mom-2* translation in early embryos, as early as the 1-cell stage based on a 3'UTR reporter (Oldenbroek et al., 2013). In our manuscript, we identified *mom-2* as a SPN-4-associated transcript. We did not analyze *mom-2* transcript levels in either study.

7. Reviewer #3. "The experiments shown in Fig. 1 (IP experiments that deine SPN-4-bound transcripts) are foundational yet lack a critical control."

We thank the reviewer for pointing out this deficiency. We conducted control experiments to examine non-specific binding of mRNA to the affinity matrix containing monoclonal anti-FLAG antibodies. As a control for the specificity of the SPN-4 immunopurifications, we conducted purifications from 5 ml of extract prepared from JK560 *fog-1(q253ts)* I and DG4398 *fog-1(q253ts)* I;*spn-4(tn1699[spn-4::gfp::tev::3xflag])* V using anti-FLAG antibodies. cDNA was prepared from the purified mRNA in two technical replicates. PCR was conducted using Q5 High-Fidelity DNA dilutions of the cDNA synthesized from the immunopurified mRNA. The 19 SPN-4-associated mRNAs (Table S1) tested were: *lin-41*, *chs-1*, *cbd-1*, *mex-3*, *puf-5*, *zif-1*, *nos-1*, *nos-2*, *egg-1*, *egg- 2*, *oma-1*, *oma-2*, *puf-3*, *puf-11*, *gfat-1*, *gfat-2*, *cpg-1*, *cpg-2* and *cpg-3*. Six controls for abundant mRNAs that were not enriched in the SPN-4 immunopurifications (Table S1) were: *ama-1*, *eef1A.1*, *rack-1*, *tbb-2*, *vit-2* and *vit-6*. The results, now shown in Fig. S3, conirm the specificity of the immunopurifications. For this control experiment, we conducted RT-PCR instead of RNA-seq because only small amounts of non-specifically bound mRNA are expected to be recovered when control extracts that do not contain SPN-4::GFP::TEV::3xFLAG are applied to the anti-FLAG Dynabeads, which was what was observed. However, we did recover what appears to be more mRNA for the abundant control transcripts in the experimental than the control, likely due to non-specific absorption of mRNA to the eluting RNPs. Importantly, for these control transcripts, our RNA-seq data shows these transcripts are not enriched over the input (Table S1 and Fig. S3B). Consistent with these indings, of the 100 most abundant mRNAs in the extract, only 5 exhibit significant enrichment in the SPN-4 immunopurifications (4-fold, P<0.05; Table S1; *cbd-1*, *cpg-1*, *cpg-2*, *vig-1*, and *ndk-1*). All of these transcripts were described as a being more abundant in oocytes as compared with 1-cell embryos (Stoeckius et al., 2014).

8. Reviewer #3. "Fig. 2: The plot shown should be replaced with that in Fig. S3E and the behavior of each lineage should be shown separately. The number of genes in each category should be indicated in the figure. This figure should also show a graph for all maternal mRNAs degraded in that time frame for comparison, to make it clear that SPN-4-bound mRNAs are a fraction of that cohort. Each plot should show the data for each gene (scatter plot style) - rather than the average for that gene group - to show the variance within each group. Are there

© 2026. Published by The Company of Biologists under the terms of the Creative Commons Attribution License (https://creativecommons.org/licenses/by/4.0/).

SPN-4 bound mRNAs that are not degraded? Is the decay behavior of SPN-4-bound transcripts similar to that observed for most maternal mRNAs?
The no-association mRNA category exhibit very low starting levels? This seems odd. The SPN-4 associated mRNAs in contrast are among the most abundant? Does that suggest that association with SPN-4 stabilizes maternal mRNAs in oocytes or zygotes?"

Thank you for your critique and guidance. We have prepared a new Fig. 2, which plots the dynamics of gene expression, grouped according to their association with SPN-4, LIN-41, and OMA-1. The number of genes in each category is now indicated. To better illustrate the dynamics across the time course, mean Z-scores of transcript abundance in reads per kilobase of transcript per million mapped reads (RPKM) are now plotted in lieu of the mean of RPKM values for dynamically changing genes (from Tintori et al., 2016). This is a more accurate way of illustrating dynamics across a time course, given the wide range of expression levels endemic to RNA-seq data. To better illustrate the variance across each transcript, Fig. S5B now shows the same data plotted as transparent line plots for each transcript.

To illustrate the fraction of SPN-4-associated transcripts that are not degraded and the degree to which SPN-4-associated transcripts difer from other transcripts, Fig. S6D now shows categorical bar plots illustrating the representation of each Cluster Set among SPN-4-associated and -unassociated transcripts.

Regarding the observation that the non-association category exhibits very low starting levels: Yes, this observation is correct and reflects that SPN-4, LIN-41, and OMA-1 are more likely to associate with maternal mRNAs than zygotic mRNAs.

Regarding the observation that SPN-4-associated transcripts tend to have high expression levels, there may be a bias towards this trend, but it is slight. To illustrate this, Figure 1D shows the high- intensity transcripts along the right side of the x-axis with SPN-4-associated transcripts colored in "red". One can observe that there are still many "black" transcripts with high intensity. Lower intensity transcripts are less likely to be identified as SPN-4 associated by virtue of the statistical thresholds that require a fold-change-increase greater than some metric of variance.

9. Reviewer #3. Fig. 3D: Most transcripts exhibit higher levels in spn-4 at the very first stage tested (2-cell). What is the evidence that SPN-4 acts in embryos, as opposed to oocytes or even an earlier stage during germline development?

Thanks for raising this issue. As mentioned above, Fig. 4 and Fig. S9 now compare *chs- 1* and *lin-41* transcript levels in oocytes from the wild type and *spn-4(tm291)* null mutants. No significant diferences were observed in transcript numbers.

II. Critiques Requiring Textual Changes, Presentational Elements, or Clarification

1. Reviewer #1. Data presented in Figure 3C for chs-1 and cpg-2 smFISH are duplicate images (both WT and spn-4 mutants). I imagine this represents a simple and innocent oversite. Please provide the correct images and double check that there are no other duplicated images throughout the figures (I did not find any).

Actually, this is not the case. These are not duplicate images, but instead the probes are labeled with different fluorophores and imaged within the same embryo. To remove any confusion, we have replaced these images with unique embryos.

2. Reviewer #1. Quantification of the mRNA abundance in various mutant backgrounds is based on fluorescent intensity of smFISH staining. I appreciate that many of these RNAs may be low abundance, but orthogonal methods for quantifying mRNA levels, such as qRT- PCR, RNA-seq and/or northern blot would greatly bolster the strength of the conclusions. This does not have to be done for every transcript analyzed, but a couple of example mRNAs would go a long way.

© 2026. Published by The Company of Biologists under the terms of the Creative Commons Attribution License (https://creativecommons.org/licenses/by/4.0/).

Our mRNA analyses are not based on fluorescence intensity. These are quantitative smFISH data in which individual transcripts are counted digitally. smFISH is widely regarded as the gold standard for obtaining absolute counts of individual mRNA molecules at single- molecule resolution (Chen et al., 2017). smFISH directly measures the transcripts that are present, whereas qRT-PCR makes indirect measurements after amplification. Further, spatial resolution is lost in qRT-PCR experiments. An important part of our analysis is comparing transcript numbers in oocytes to early embryos-it would be problematic to dissect oocytes from gonads, as spatial information regarding their position in the gonad would be lost.

3. Reviewer #1. Along the same lines, the authors often express mRNA levels as number of transcripts. Were spike-ins or other controls run to ensure that fluorescence always tightly correlated with transcript number?

The smFISH protocol and analytical pipeline yields number of transcripts as a digital output. The spots are assessed based on their size and intensity metrics (Imbert et al., 2022). Even clusters of mRNA can be deconvolved to individual numbers based on their size and intensity metrics. smFISH utilizes 20-40 short probes per transcript so that single- molecule spots exhibit a narrow intensity distribution corresponding to multiple fluorophores per RNA molecule. We confirmed that the cytoplasmic spots form a unimodal, tight intensity distribution in keeping with the fish-quantv2 (bigfish) best practices. We also observed consistent counting across multiple analysis pipelines.

4. Reviewer #1. In Figure 4B, the lin-41 mRNA signal for the Δ777-1054 deletion looks lower throughout the embryo in the 4-cell image provided. In addition, the signal appears in large puncta. Do the authors have an explanation for this pattern? Is this typical for this specific construct?

Thank you for pointing this out. The prior image in Figure 4B was incorrectly scaled. New images with properly scaling are included in what is now Fig. GB. When SPN-4-dependent maternal mRNA clearance is disrupted, we frequently observe the transcripts in granules within germline blastomeres. The nature of these granules and their biological significance is something we are actively working on.

5. Reviewer #1. This may be beyond the current scope of the paper, but pulse-labeling experiments designed to measure mRNA half-lives +/- spn-4 would enhance the conclusions of the story.

This is an interesting suggestion, but we are uncertain how this could be accomplished in practice. Recently, Peng et al. (2024) published an exciting and informative study in which they examined the decay rate of zygotic mRNAs. They treated primary cultures of *C. elegans* embryonic cells with the transcription inhibitor Actinomycin D and measured mRNA decay rates genome-wide using a combination of bulk and single-cell RNA-seq. However, we are examining the turnover of maternal transcripts. By and large, maternal mRNAs are transcribed in the pachytene region of the gonad, so the methods used by Peng et al. would not appear to be transferable to maternal mRNAs. In principle, one might hand pick embryos of precise stages and conduct qRT-PCR measurements, but it is not clear that would be an improvement over our smFISH data. The SPN-4-associated mRNAs that we have studied appear to have biphasic half-lives. There is a rapid SPN-4-dependent decay commencing after fertilization as well as a slower SPN-4-independent decay. In the future, we hope that methods for visualizing transcripts *in vivo* in a time-resolved fashion will enable us to address this very interesting and important question.

6. Reviewer #2. In figure 4B, when there is less degradation of the lin41 RNA, particularly in the Med and Lg foxΔ, it seems like there is an accumulation of RNA in P-granules, which is potentially interesting. This is also true for *chs-1* RNA in figure GB. Could this potential concentration of RNA into P-granules be concentration

© 2026. Published by The Company of Biologists under the terms of the Creative Commons Attribution License (https://creativecommons.org/licenses/by/4.0/).

dependent? The authors should potentially comment on this or try a P-granule marker to confirm this, but this is not necessary for this publication.

Thank you for the astute observation and the comment. The nature of these RNP granules in the germline blastomeres is something we are actively studying. The granules may yield insight into how SPN-4-dependent mRNA clearance is attenuated or prevented in germline blastomeres (Fig. 5).

7. Reviewer #2. In figure 4, even though there is a clear elevation of lin41 RNA in the fox deletions compared to WT, there is still a substantial decrease from the 2-4 cell stage. Is this due to normal turnover? Or due to another mechanism of degradation? The authors should comment on this.

Thank you for bringing up this issue. Since this decrease doesn't happen when SPN-4 is removed (now Fig. 3), this observation suggests that other sequences or factors may be involved. We have added the following statement to what is now the legend to Fig. GD. "The decrease in *lin-41* mRNA levels in the RbFox site deletions might be mediated by other sequences or factors that work with SPN-4."

8. Reviewer #2. In figure 5B, I assume that the right side is the 4-cell stage (it is labelled as the 2-cell stage, identical to the left side, so the labelling should be fixed). If so, there is now no increase in lin41 RNA in the sm fox deletion at the 4-cell stage like there was in figure 4C. Also, if there is no increase in this experiment, the image in 5A does not appear to be representative since it looks like there is an increase. The authors should probably comment on this.

Thank you for pointing this out. There were two issues here. The first was that in trying to meet the size limitations of the submitted pdf required by the *Development* website, an earlier version of the figure was included by mistake. The second was that there was an issue with the scaling of the images. These errors have been fixed.

9. Reviewer #2. I don't think figure GF is referenced in the text.

Thank you for catching this. This is now Fig. 8F and we have referred to it.

10. Reviewer #2. It would be helpful if the authors provided more introduction of the CCR4-NOT deadenylase complex, including LET711, CCF1 and CCR4

Thanks! We have done so. Lines 331-335 read, "In addition, we isolated 10 affecting the CCR4-NOT deadenylase complex, a conserved multi-subunit enzyme containing the LET-711 scaffold and the CCF-1 and CCR-4 deadenylases (Nousch et al., 2013). The CCR-NOT deadenylase complex is central to mRNA metabolism; it deadenylates mRNA to promote mRNA decay or translational repression (Caulier et al., 2025)."

11. Reviewer #2. I believe that figure 9F is not cited in the text.

Thanks! We now refer to what is now Fig. 11F.

12. Reviewer #2. In figure 10, depletion of LET711 appears to block mRNA degradation, while depletion of CCF1 seems to more delay degradation. The authors should comment on this. Also, there is no corresponding image of CCR-4. Ideally this would be shown.

Thanks! This difference is likely due to the fact that there are two deadenylases in the complex. We point out that CCR-4 is a second deadenylase in the complex, and we showed that a *ccr-4* null mutations enhances the phenotype of a *ccf-1* reduction-of-function allele. We have included an image showing *lin-41* and *chs-1* smFISH in *ccr-4(tm1312)* null mutants in Fig. S17.

© 2026. Published by The Company of Biologists under the terms of the Creative Commons Attribution License (https://creativecommons.org/licenses/by/4.0/).

13. Reviewer #2. I hate to even suggest this, because of all the work that has already been done, but a simple model figure would be helpful.

Great suggestion! We included a model figure (Fig. 13).

14. This question is not relevant to this current paper or the review, but I think it would be interesting to ask, does this system only affect maternal RNAs? Or does it also affect any siRNAs?

Interesting question! We do not know whether SPN-4 binds siRNAs because the sequencing technology employed would not have captured short RNA molecules. However, there is precedent for RNA-binding proteins recruiting siRNA to compete with or facilitate ARGONAUTE binding. To ask whether SPN-4-associated mRNAs that escape SPN-4 maternal mRNA clearance are degraded by CSR-1 siRNAs in the next wave of maternal mRNA decay, we assessed the overlap between our set of SPN-4-associated mRNAs and those that depend on CSR-1 for clearance (Quarato et al., 2021). Though the two cohorts did overlap with higher frequency than expected by chance, the absolute numbers of transcripts that were degraded by both pathways represented a minority of both cohorts.
We felt those findings failed to fit into this already long manuscript and would better serve a manuscript that more directly addresses the SPN-4 siRNA connection.

15. Reviewer #3. "The authors should also show expression of SPN-4 in the feminized worms used for the IP."

We have added these images to Fig. S1. The images also highlight the fact that SPN-4 is more abundant in embryos than in oocytes.

16. Reviewer #3. Why was the spn-4 GFP introduced on a balancer chromosome?? Balancer chromosomes are usually used to suppress recombination but that does not seem the purpose there. Does the balancer chromosome also have a lethal mutation to prevent the propagation of spn-4::GFP homozygotes?

SPN-4::GFP was introduced onto the *tmC3* balancer chromosome (Dejima et al., 2018) so that large numbers of embryos could be isolated for smFISH analyses such that embryos without GFP fluorescence must have been produced by *spn-4(tm291)* homozygotes and lacked maternal *spn-4(+)* activity. This *tmC3* derivative serves to suppress recombination in the region of chromosome V where *spn-4* maps and avoids the production of aneuploid embryos that reciprocal translocations produce (e.g., *nT1* and its derivatives). *tmC3* homozygotes are not lethal but are sickly and produce few progeny. Please note that in Fig. 3, the smFISH data compare data from the wild type (strain N2) to embryos lacking maternal SPN-4::GFP [e.g., *spn-4(tm291)* homozygous null mutant embryos]. Using hand-dissected embryos from *spn-4(tm291)* null mutants, we validated the larger throughput approach using the balancer chromosome (unpublished results).

17. Fig. 8: The inference that CCR4-NOT are candidate effectors of SPN-4 mediated degradation is not supported by data showing direct interactions between the two or a specific effect of CCR4-NOT on spn-4-bound transcripts. CCR4-NOT has already been reported to be the main deadenylase in the C. elegans germline, so it is likely to affect all maternal mRNAs?

We conducted a genetic screen for genes that function together with *spn-4* (e.g., the genetic screen for dominant suppressors of *puf-3111Δ* maternal effect lethality). In addition to recovering a strong loss-of-function *spn-4* allele, we recovered many *let-711* alleles and a *ccf-1* allele (Fig. 11). We used the auxin-inducible degradation system to deplete LET-711 and CCF-1 and observed a marked effect on *lin-41* and *chs-1* transcript levels in early embryos and only modest effects on the *set-3* control (~1.5-fold). Importantly, the viable and fertile *ccf-1(tn2168)* allele appears to specifically affect *chs-1* and *lin-41* transcript levels and not the *set-3* control (Figs 12, S17 and S18). The reviewer is correct in pointing out that we have yet to define direct interactions between SPN-4 and the CCR4-NOT complex. We are planning to address this issue in future studies. The proteomic data we do have suggest that the CCR4-

© 2026. Published by The Company of Biologists under the terms of the Creative Commons Attribution License (https://creativecommons.org/licenses/by/4.0/).

NOT complex recruitment happens in the embryo, not the oocyte. Our current hypothesis is that intrinsically disordered regions near the SPN-4 C-terminus mediate multivalent interactions with the CCR4-NOT complex.

18. Reviewer #3. All figures: The authors should adopt a consistent color scheme throughout and report the data using superplots where individual data points are shown (instead of averages) and color coded to show replicates where applicable.

We have used a consistent color scheme and have presented all data and replicates in the figures, supplemental figures, and supplemental tables.

Second decision letter

MS ID#: dev.205295R1

MS TITLE: The SPN-4 Rbfox RNA-binding protein selects maternal mRNAs for CCR4-NOT-dependent clearance in early Caenorhabditis *elegans embryos*

AUTHORS: Caroline A. Spike, Dylan M. Parker, Tatsuya Tsukamoto, Naly Torres-Mangual, Erika C. Tsukamoto, Karissa Coleman, Micah D. Gearhart, David Greenstein and Erin Osborne Nishimura

Dear Dr Osborne Nishimura,

I have now received all the referees' reports on the above manuscript, and have reached a decision. The referees' comments are appended below.

As you will see, the manuscript was re-evaluated by two reviewers, since Reviewer 1 was not available to assess the revision. Reviewer 2 in this round of review, who was Reviewer 3 in the previous round, again notes a point also raised previously and reflected in the manuscript: in the absence of spn-4, GFP::LIN-41 is elevated relative to wild type, as shown in Figure S12D of the revised submission. The reviewer recommends that this observation be incorporated into the overall conclusion, as it also supports a model in which SPN-4 regulates GFP::LIN-41 translationally particularly given the complexity of the relevant cell type and developmental context. The authors should therefore acknowledge the broader role of SPN-4, which likely includes translational repression, in reaching their final conclusions. I thus ask for an additional round of revision which addresses the points raised by both reviewers 1 and 2 from this review cycle. Please send us a point-by-point response indicating your plans for addressing the referees' comments, and we will look over this and provide further guidance.

Reviewer 2

Advance summary and potential significance to field

Overall, this is a truly outstanding paper with tons of high quality data that is well described and beautifully presented. As described in the summary above, the data have major mechanistic implications for our understanding of how maternal mRNAs are degraded.

Comments for the author

In figure 3E, the figure legend should indicate what the dashed lines represent.

Other than that, the paper is suitable for publication.

Reviewer 3

© 2026. Published by The Company of Biologists under the terms of the Creative Commons Attribution License (https://creativecommons.org/licenses/by/4.0/).

Advance summary and potential significance to field

This manuscript identifies mRNAs that associate with the Rbfox-related RNA-binding protein SPN-4 in C. elegans oocytes and documents a correlation between SPN-4 association and mRNA decay in early embryos. The authors estimate that SPN-4 may be responsible for the turnover of approximately 11% of maternal mRNAs. Using smFISH, they confirm that several SPN-4-associated mRNAs are stabilized in spn-4 mutants. The study also identifies Rbfox-binding motifs in the 3'UTRs of target mRNAs that are required for SPN-4-dependent clearance and that bind the SPN-4 RRM in vitro. A genetic screen identifies CCR4-NOT deadenylase components as factors that work with SPN-4 in this process. The experimental work is extensive and of generally high quality.

However, a central claim of the paper" that SPN-4 directly promotes mRNA decay" is not adequately supported and is contradicted by the authors' own protein-level data. I raised this concern in the first round of review; the new data provided in response actually strengthen rather than resolve this concern, as detailed below.

Comments for the author

1. The authors' own protein data contradict the central claim that SPN-4 directly promotes mRNA decay.

In the first round of reviews, I raised the possibility that SPN-4's effect on mRNA stability could be indirect and secondary to its documented role as a translational repressor. In response, the authors examined GFP::LIN-41 protein levels in spn-4 mutant backgrounds (new Fig. 9) and state (line 298) that "GFP::LIN-41 protein was not markedly elevated in spn-4 null or small FoxΔ backgrounds." This statement lacks quantitative support in the main figure. The quantitative data presented in Fig. S12B tell a different story.
The Fig. S12B data show that GFP::LIN-41 protein levels are elevated in spn-4 mutant embryos relative to controls and appear to slowly increase over time. Furthermore, in sel-10 single mutant embryos, LIN-41 starts at high levels but declines over time. This is expected if some protein turnover still occurs (sel-10 only attenuates ubiquitin-mediated degradation; Fig. 9 confirms that GFP::LIN-41 levels are still lower in embryos compared to oocytes in the sel-10 background) while SPN-4 continues to suppress new translation. Most strikingly, in the sel-10; spn-4 double mutant, high LIN-41 levels remain stable over time. This pattern — elevated and stable protein only when both protein degradation and translational repression are removed — is entirely consistent with a model in which SPN-4 represses translation of lin-41 mRNA and the observed mRNA decay is a secondary consequence of translational repression.

These data directly contradict the authors' claim and provide evidence supporting the alternative model I proposed in my original review. The manuscript should not claim that SPN-4 directly promotes mRNA decay when the protein-level data are more parsimoniously explained by an indirect effect through translational repression. At minimum, the authors must accurately describe what Fig. S12B shows and acknowledge that their data do not exclude — and are in fact consistent with — translational repression as the primary function of SPN-4.
This concern is reinforced by the phenotypic evidence: spn-4 mutants are embryonic lethal and exhibit spindle orientation defects at the 2-cell stage — phenotypes that are NOT observed in mutants where maternal mRNA decay is blocked. If SPN-4's primary function were to promote mRNA decay, loss of SPN-4 and loss of the decay machinery should produce more similar phenotypes. The simplest interpretation is that SPN-4 has essential functions beyond promoting mRNA decay, most likely translational repression, that account for the more severe phenotype.

2. The conclusion that SPN-4-dependent clearance is preferentially activated in somatic blastomeres is not adequately supported.

The authors examine the decay pattern of lin-41 mRNA in somatic versus germline lineages and conclude that "SPN-4-dependent clearance is preferentially activated in somatic blastomeres and attenuated in germline blastomeres." This conclusion is complicated by several issues. First, mRNAs associated with P granules are preferentially partitioned into P blastomeres at each division and therefore are expected to be present at higher concentrations in these cells even if subject to the

© 2026. Published by The Company of Biologists under the terms of the Creative Commons Attribution License (https://creativecommons.org/licenses/by/4.0/).

same rate of decay. The apparent somatic preference in clearance could reflect asymmetric mRNA partitioning rather than differential decay rates. Second, Fig. 2B suggests that SPN-4-bound mRNAs do undergo decay in the P lineage, albeit perhaps at a slower rate. Third, the authors did not examine mRNA levels in P blastomeres in spn-4 mutant embryos, which would be necessary to determine whether the apparently slower decay in germline cells is truly SPN-4-independent. The authors should at minimum acknowledge these caveats and temper their conclusion.

Minor Points

3. CCR4-NOT suppressor screen.

The identification of CCR4-NOT deadenylase components through a genetic screen for suppressors of puf-3/11Δ maternal-effect lethality is an interesting result. The authors should clarify which suppressor mutations were recovered in the heterozygous state (i.e., as dominant suppressors). Additionally, the requirement for CCR4-NOT in degradation of SPN-4 target mRNAs is consistent with prior studies showing that CCR4-NOT is the primary deadenylase in C. elegans embryos (Nousch et al., 2013). This finding alone does not distinguish between a model in which SPN-4 directly recruits CCR4-NOT to target mRNAs versus one in which CCR4-NOT acts downstream of translational repression to degrade untranslated mRNAs.

4. Lines 471-475: the phenotypic discrepancy requires direct acknowledgment.

This passage attempts to reconcile the observation that spn-4 mutant embryos exhibit severe phenotypes (embryonic lethality, abnormal spindle orientation) not observed in mutants where maternal mRNA decay is delayed. The authors' conclusion that "SPN-4-dependent mRNA clearance is required for sculpting the embryonic transcriptome" does not address this discrepancy. If SPN-4's role were limited to promoting mRNA decay, blocking mRNA decay should phenocopy spn-4 loss, but it does not. The authors should state directly that SPN-4 must have functions beyond promoting mRNA clearance, consistent with its documented role as a translational repressor.

Second revision

<u>Author response to reviewers' comments</u>

We thank the reviewers and the editor for the constructive suggestions. We have addressed all points raised. We have included a pdf file of the manuscript in which changes in response to the reviewers' comments are highlighted.

Reviewer #2

1. Overall, this is a truly outstanding paper with tons of high quality data that is well described and beautifully presented. As described in the summary above, the data have major mechanistic implications for our understanding of how maternal mRNAs are degraded.
   We thank the reviewer for the positive assessment of our work.

2. In figure 3E, the figure legend should indicate what the dashed lines represent. Other than that, the paper is suitable for publication.
   We have added a sentence to the figure legend, as follows, "The dotted lines indicate cutoff threshold values such that the genes shown in the upper right quadrant are both highly enriched and statistically significant." We have also improved the labelling of the x-axis.

Reviewer #3

1. The authors' own protein data contradict the central claim that SPN-4 directly promotes mRNA decay.

© 2026. Published by The Company of Biologists under the terms of the Creative Commons Attribution License (https://creativecommons.org/licenses/by/4.0/).

In the first round of reviews, I raised the possibility that SPN-4's effect on mRNA stability could be indirect and secondary to its documented role as a translational repressor. In response, the authors examined GFP::LIN-41 protein levels in spn-4 mutant backgrounds (new Fig. 9) and state (line 298) that "GFP::LIN-41 protein was not markedly elevated in spn-4 null or small FoxΔ backgrounds." This statement lacks quantitative support in the main figure. The quantitative data presented in Fig. S12B tell a different story.

mRNA and the observed mRNA decay is a secondary consequence of translational repression.

These data directly contradict the authors' claim and provide evidence supporting the alternative model I proposed in my original review. The manuscript should not claim that SPN-4 directly promotes mRNA decay when the protein-level data are more parsimoniously explained by an indirect effect through translational repression. At minimum, the authors must accurately describe what Fig. S12B shows and acknowledge that their data do not exclude — and are in fact consistent with — translational repression as the primary function of SPN-4. This concern is reinforced by the phenotypic evidence: spn-4 mutants are embryonic lethal and exhibit spindle orientation defects at the 2-cell stage — phenotypes that are NOT observed in mutants where maternal mRNA decay is blocked. If SPN-4's primary function were to promote mRNA decay, loss of SPN-4 and loss of the decay machinery should produce more similar phenotypes. The simplest interpretation is that SPN-4 has essential functions beyond promoting mRNA decay, most likely translational repression, that account for the more severe phenotype.

We respectfully disagree with the reviewer's assessment that Figure S12D "tells a different story." This is likely because we didn't do an effective job in fully describing the results and explaining the data in the interest of space limitations. We have revised the paragraph describing these data as follows (revised portions are underlined and we have attached Figure S12 and its legend for ease of reviewing by the Editor):

"The observation that SPN-4 functions through the Rbfox motif to reduce *lin-41* transcript levels ~10-fold in somatic blastomeres—together with evidence that it impacts early embryonic gene expression (Gomes et al., 2001; Huang and Hunter, 2015; Huang et al., 2002; Ogura et al., 2003) and can act as a translational repressor (Jadhav et al., 2008; Oldenbroek et al., 2012, 2013)—prompted us to assess *FoxΔ* and *spn-4* null mutant backgrounds for effects on GFP::LIN-41 protein levels in early embryos. Upon meiotic maturation onset, LIN-41 undergoes SCF^{SEL-10} E3 ubiquitin ligase-mediated degradation (Spike et al., 2018; Spike et al., 2022). Thus, we also analyzed the viable, fertile *sel-10(ok1632)* null background, where ubiquitin-mediated degradation of LIN-41 is prevented but maternal *lin-41* mRNA clearance remains unaffected (Fig. S12A-C). There were no increases in GFP::LIN-41 levels in *FoxΔ* mutants relative to controls (Fig. S12D). We observed a modest increase in GFP::LIN-41 levels in *spn-4* null mutant embryos relative to the wild type (a ~1.5-fold increase by the 24-cell stage; Figs 9 and S12D). The magnitude of this change was small relative to the increases in *lin-41* mRNA levels observed in *spn-4* null mutant embryos (a ~10- fold increase by the 8-cell stage; Fig. 3D). This observation suggests that the translational efficiency per transcript is lower in *spn-4* mutants than in the wild type. In the *spn-4; sel-10* null background, we unexpectedly observed slightly lower GFP::LIN-41 levels than observed in the *sel-10* background. GFP::LIN-41 levels in the *spn-4; sel-10* background did not appear to change as embryos developed, although a modest~30% decrease was observed in *spn-4(+); sel-10* embryos by the 24-cell stage (Figs 9 and S12D). Taken together, these observations suggest that LIN-41 is not robustly translated in the early embryo when SPN-4-mediated *lin-41* transcript clearance is disrupted. However, they do not rule out the possibility that SPN-4 represses LIN-41 translation. Indeed, the final levels of LIN-41 likely result from the combined impact of SPN-4 on translation efficiency and mRNA

© 2026. Published by The Company of Biologists under the terms of the Creative Commons Attribution License (https://creativecommons.org/licenses/by/4.0/).

stability, two processes that may be linked."

To acknowledge the broader role that SPN-4 plays, we have added the following sentence to the first paragraph of the Discussion as follows:

"Although our data do not exclude the possibility that SPN-4 can function as a translational repressor, we did not observe markedly elevated levels of LIN-41 protein in *spn-4* null mutant embryos or after disrupting the SPN-4-binding site in the *lin-41* 3'UTR, despite the fact that *lin-41* transcript numbers are substantially elevated (~10- fold) in these genetic backgrounds."

We appreciate the comment regarding the difference between null phenotypes of *spn-4* and components of the CCR4-NOT complex. We failed to include and discuss a key reference. We have modified the second paragraph of the Discussion as follows (the underlined text is new):

"Our genetic and smFISH experiments suggest that the CCR4-NOT deadenylase complex is the major effector of SPN-4-dependent maternal mRNA clearance. A genetic screen designed to recover mutant alleles of genes that function with SPN-4 isolated alleles of *let-711* and *ccf-1*. Because components of the CCR4-NOT complex are required for viability, we utilized the AID system to deplete LET-711 and CCF-1 late in oogenesis and found that these treatments blocked the elimination of *lin-41* mRNA and *chs-1* mRNA. Strong loss-of-function mutations in *let-711*, which encodes the largest scaffolding subunit of the CCR4-NOT deadenylase complex, exhibit larval arrest and sterile phenotypes (Stewart et al., 1998; DeBella et al., 2006), consistent with its role as the main deadenylase in *C. elegans* (Nousch et al., 2013). Homozygous *let-711* null mutants complete embryogenesis using maternally supplied protein and mRNA. Interestingly, the *let-711(it150)* reduction-of-function mutation exhibits a spindle-positioning defect in the early embryo (DeBella et al., 2006), similar to *spn-4* mutants (Gomes et al., 2001). This observation is consistent with the conclusion that the CCR4- NOT complex functions in the SPN-4 pathway for maternal mRNA clearance. Future work will be needed to address whether SPN-4 directly associates with the CCR4-NOT complex and recruits it to mRNA to promote decay. Our analyses in *C. elegans* are consistent with findings from other systems that the CCR4-NOT deadenylase complex is a major effector of maternal mRNA clearance (Ma et al., 2015; Mishima and Tomari, 2016; Semotok et al., 2005; Sha et al., 2018; Soeda et al., 2023; Yu et al., 2016). The CCR4-NOT deadenylase complex also participates in translational regulation by microRNAs illustrating how multiple mRNA targeting triggers can deploy the complex to promote RNA decay or translational repression (Braun et al., 2011; Chekulaeva et al., 2011; Fabian et al., 2011). In *Drosophila*, *Xenopus* and zebrafish, zygotically expressed microRNAs mediate a late phase of maternal mRNA clearance (Bushati et al., 2008; Giraldez et al., 2006; Lund et al., 2009)."

2. The conclusion that SPN-4-dependent clearance is preferentially activated in somatic blastomeres is not adequately supported.

The authors examine the decay pattern of lin-41 mRNA in somatic versus germline lineages and conclude that "SPN-4-dependent clearance is preferentially activated in somatic blastomeres and attenuated in germline blastomeres." This conclusion is complicated by several issues. First, mRNAs associated with P granules are preferentially partitioned into P blastomeres at each division and therefore are expected to be present at higher concentrations in these cells even if subject to the same rate of decay. The apparent somatic preference in clearance could reflect asymmetric mRNA partitioning rather than differential decay rates. Second, Fig. 2B suggests that SPN-4-bound mRNAs do undergo decay in the P lineage, albeit perhaps at a slower rate. Third, the authors did not examine mRNA levels in P blastomeres in spn-4 mutant embryos, which would be necessary to determine whether the apparently slower decay in germline cells is truly SPN-4-

© 2026. Published by The Company of Biologists under the terms of the Creative Commons Attribution License (https://creativecommons.org/licenses/by/4.0/).

independent. The authors should at minimum acknowledge these caveats and temper their conclusion.

The reviewer's point about asymmetric partitioning in the P lineage is well taken; however, this point does not apply to the division of AB. We show that when AB divides, the ABa and ABp sisters contain vastly less *lin-41* mRNA than its parent. Thus, we have modified the text as follows (the underlined portion is new):

"To test whether SPN-4-dependent mRNA clearance is spatially regulated in the embryo, we reanalyzed our *lin-41* smFISH data at the 2- and 4-cell stages with cell- specific resolution to assess *lin-41* transcript concentration in AB, P1, ABa, ABp, EMS and P2 cells (Fig. 5). *lin-41* transcripts were significantly less concentrated in the somatic blastomere AB than in its germline sister P1, whereas the *set-3* mRNA controls showed no marked AB–P1 asymmetry. After AB divided, *lin-41* transcripts declined sharply in ABa and ABp synchronously, indicating robust clearance in the AB lineage. By contrast, the P1 daughters displayed asymmetric *lin-41* levels, with the somatic blastomere EMS containing substantially fewer transcripts than the germline blastomere P2. The difference in *lin-41* transcript numbers observed between somatic and germline blastomeres (e.g., between AB vs. P1 and between EMS vs. P2) could be due to asymmetric mRNA partitioning of P-granules to germline blastomeres; however, this possibility does not apply to the division of AB. These data are consistent with the possibility that SPN-4-dependent clearance is preferentially activated in somatic blastomeres. Consistent with this view, the *nanos* homologs *nos-1* and *nos-2*, which we identified as SPN-4-associated transcripts (Table S1), coexist with SPN-4 in germline blastomeres (Jadhav et al., 2008; Subramaniam and Seydoux, 2003). We speculate that additional factors in germline blastomeres modulate SPN-4 target selection or activity to preserve *nos-1*, *nos*-2 and other germline mRNAs."

3. CCR4-NOT suppressor screen.
The identification of CCR4-NOT deadenylase components through a genetic screen for suppressors of puf-3/11Δ maternal-effect lethality is an interesting result. The authors should clarify which suppressor mutations were recovered in the heterozygous state (i.e., as dominant suppressors). Additionally, the requirement for CCR4-NOT in degradation of SPN-4 target mRNAs is consistent with prior studies showing that CCR4-NOT is the primary deadenylase in C. elegans embryos (Nousch et al., 2013). This finding alone does not distinguish between a model in which SPN-4 directly recruits CCR4-NOT to target mRNAs versus one in which CCR4-NOT acts downstream of translational repression to degrade untranslated mRNAs.

We have clarified that all the mutations isolated in the genetic screen were isolated as dominant suppressors. The only mutation from the EMS screen that can be grown as a homozygote is the *let-711(tn2088* P1271L*)* mutant. It is shown as boxed in Figure 11 to indicate that property as explained in the figure legend. To add clarity, we added the following statement in the Materials and Methods section:

"All of the suppressor mutations were isolated in the F1 generation following mutagenesis and exhibited dominance. The *let-711(tn2088* P1271L*)* mutation could be maintained as a homozygous strain."

As mentioned above, we addressed the issue of CCR4-NOT recruitment in the Discussion (see point #1 above).

4. Lines 471-475: the phenotypic discrepancy requires direct acknowledgment. This passage attempts to reconcile the observation that spn-4 mutant embryos exhibit severe phenotypes (embryonic lethality, abnormal spindle orientation) not observed in mutants where maternal mRNA decay is delayed. The authors'

© 2026. Published by The Company of Biologists under the terms of the Creative Commons Attribution License (https://creativecommons.org/licenses/by/4.0/).

conclusion that "SPN-4- dependent mRNA clearance is required for sculpting the embryonic transcriptome" does not address this discrepancy. If SPN-4's role were limited to promoting mRNA decay, blocking mRNA decay should phenocopy spn-4 loss, but it does not. The authors should state directly that SPN-4 must have functions beyond promoting mRNA clearance, consistent with its documented role as a translational repressor.

As mentioned above, there is a reduction-of-function *let-711* allele that has a spindle- positioning defect in the early embryo (DeBella et al., 2006). However, we added the following statement to this section in the Discussion (new text is underlined):

“Although we cannot exclude the possibility that the protein products of some SPN-4- associated transcripts are incompatible with embryonic development, mRNA clearance can be delayed without deleterious consequences in several mutant backgrounds. Further, *spn-4* null mutants exhibit highly pleiotropic defects that manifest within the first few cell divisions. Based on these observations, we suggest that SPN-4-dependent mRNA clearance is required for sculpting the embryonic transcriptome. Yet, as discussed above, SPN-4 must have functions beyond promoting mRNA clearance, consistent with its documented role as a translational repressor (Jadhav et al., 2008; Oldenbroek et al., 2012, 2013).”

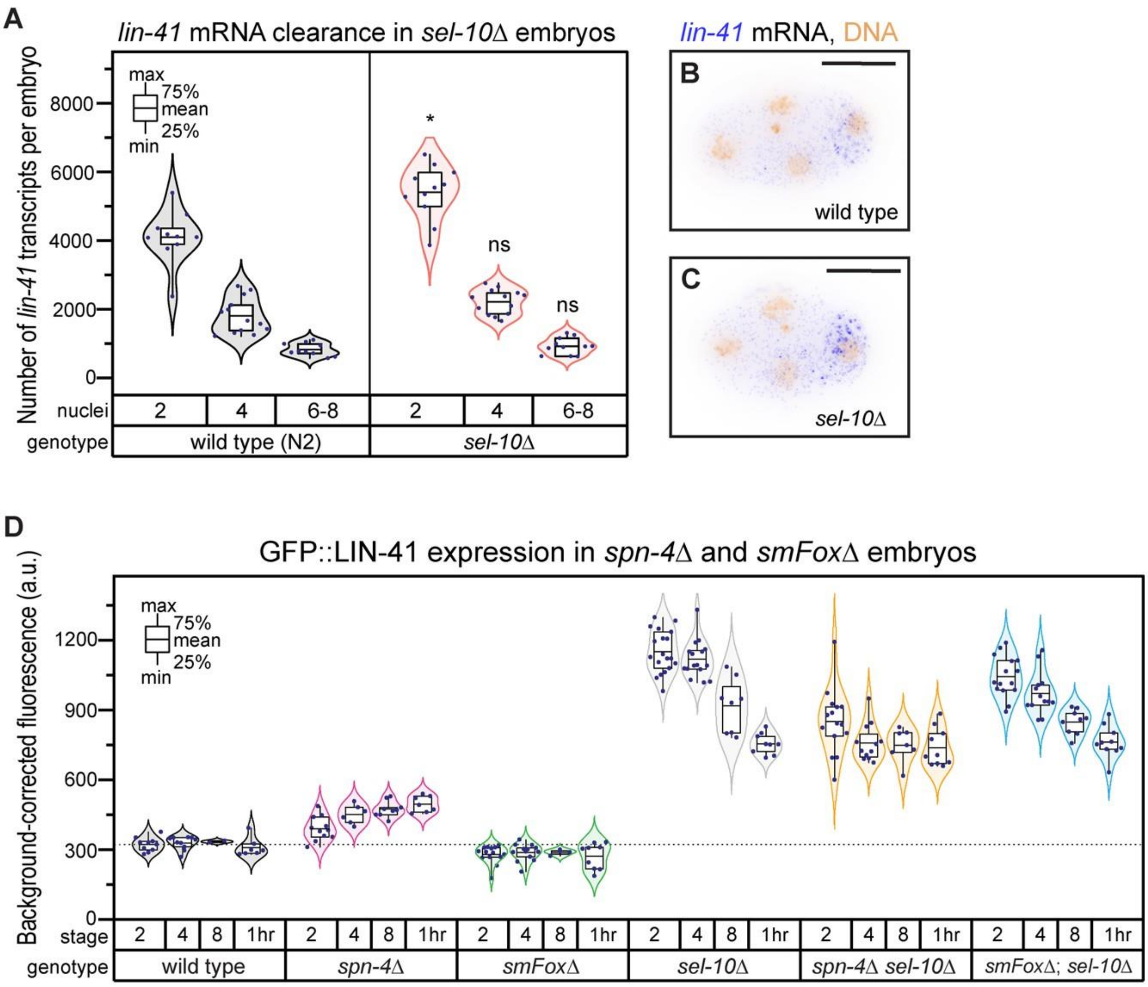

Fig. S12. A *sel-10* null mutation does not a4ect clearance of *lin-41* mRNA and analysis of GFP::LIN-41 levels in *sel-10* and smFoxΔ mutant backgrounds. (Related to Fig. 9). (A-C) smFISH data for *lin-41* in the wild type and *sel-10(ok1632)* null mutant (*sel-10Δ*) embryos. Quantitative (A) and representative smFISH (B,C) data. Bars, 20 µm. Note *lin-41* transcript levels are slightly

© 2026. Published by The Company of Biologists under the terms of the Creative Commons Attribution License (https://creativecommons.org/licenses/by/4.0/).

elevated in sel-10Δ mutants at the 2-cell stage (*P*=0.012, asterisk, using Welch's one-way ANOVA with a Games-Howell post- hoc test), however, *lin-41* transcript levels are not significantly (ns) diRerent from the wild type at the 4- and 6-8-cell stages. See Table S15 for exact *P* and n values. (D) Quantitative analysis of background-corrected GFP::LIN-41 fluorescence levels in 2-cell, 4-cell, 8-cell and "1hr"-embryos (1hr-embryos were examined 1 hr after the 2-cell stage, which corresponds approximately to the 24-cell stage in the wild type). The dotted line indicates the average fluorescence levels of all wild-type embryos, which is attributed to autofluorescence. Genotypes: wild type–DG3913 *lin-41(tn1541[gfp::s-tag::lin-41]*) I, spn-4Δ– lin-41(tn1541[gfp::s-tag::lin-41]) I; *spn-4(tm291)* V from DG4517, *smFoxΔ*–DG5779 *lin-41(tn1541[gfp::s-tag::lin-41] tn2200)* I, *sel-10Δ-DG4310 lin-41(tn1541[gfp::s-tag::lin-41]*) I; *sel-10(ok1632)* V, *spn-4D sel-10Δ-lin-41(tn1541[gfp::s-tag::lin-41]* I; *spn-4(tm291) sel-10(ok1632)* V from DG4549, *smFoxD sel-10Δ-DG6272 lin-41(tn1541[gfp::s-tag::lin-41]) tn2200)* I; *sel-10(ok1632)* V. GFP::LIN-41 expression data and n values are in Table S13.

Third decision letter

MS ID#: dev.205295R2

MS TITLE: The SPN-4 Rbfox RNA-binding protein selects maternal mRNAs for CCR4-NOT-dependent clearance in early Caenorhabditis *elegans embryos*

AUTHORS: Caroline A. Spike, Dylan M. Parker, Tatsuya Tsukamoto, Naly Torres-Mangual, Erika C. Tsukamoto, Karissa Coleman, Micah D. Gearhart, David Greenstein and Erin Osborne Nishimura

Dear Dr Osborne Nishimura,

I am happy to tell you that your manuscript has been accepted for publication in Development, pending our standard publication integrity checks.

© 2026. Published by The Company of Biologists under the terms of the Creative Commons Attribution License (https://creativecommons.org/licenses/by/4.0/).
